# Emergent Biosynthetic Capacity in Simple Microbial Communities

**DOI:** 10.1371/journal.pcbi.1003695

**Published:** 2014-07-03

**Authors:** Hsuan-Chao Chiu, Roie Levy, Elhanan Borenstein

**Affiliations:** 1 Department of Genome Sciences, University of Washington, Seattle, Washington, United States of America; 2 Department of Computer Science and Engineering, University of Washington, Seattle, Washington, United States of America; 3 Santa Fe Institute, Santa Fe, New Mexico, United States of America; The Centre for Research and Technology Hellas, Greece

## Abstract

Microbes have an astonishing capacity to transform their environments. Yet, the metabolic capacity of a single species is limited and the vast majority of microorganisms form complex communities and join forces to exhibit capabilities far exceeding those achieved by any single species. Such enhanced metabolic capacities represent a promising route to many medical, environmental, and industrial applications and call for the development of a predictive, systems-level understanding of synergistic microbial capacity. Here we present a comprehensive computational framework, integrating high-quality metabolic models of multiple species, temporal dynamics, and flux variability analysis, to study the metabolic capacity and dynamics of simple two-species microbial ecosystems. We specifically focus on detecting *emergent biosynthetic capacity* – instances in which a community growing on some medium produces and secretes metabolites that are not secreted by any member species when growing in isolation on that same medium. Using this framework to model a large collection of two-species communities on multiple media, we demonstrate that emergent biosynthetic capacity is highly prevalent. We identify commonly observed emergent metabolites and metabolic reprogramming patterns, characterizing typical mechanisms of emergent capacity. We further find that emergent secretion tends to occur in two waves, the first as soon as the two organisms are introduced, and the second when the medium is depleted and nutrients become limited. Finally, aiming to identify global community determinants of emergent capacity, we find a marked association between the level of emergent biosynthetic capacity and the functional/phylogenetic distance between community members. Specifically, we demonstrate a “Goldilocks” principle, where high levels of emergent capacity are observed when the species comprising the community are functionally neither too close, nor too distant. Taken together, our results demonstrate the potential to design and engineer synthetic communities capable of novel metabolic activities and point to promising future directions in environmental and clinical bioengineering.

## Author Summary

Microbes constantly change their environment, consuming some compounds from their surroundings and secreting others. This microbial activity plays a crucial role in many important environmental cycles, ultimately making all life possible. These processes, however, are often not accomplished by a single species but rather by a diverse community of interacting microorganisms. Characterizing these interactions and their impact is essential not only for understanding global ecosystem metabolism, but also for uncovering the tremendous potential of microbial communities in industrial, environmental, and clinical applications. In this paper, we present a computational framework for modeling, exploring, and tracking such enhanced metabolic capacities in simple two-species communities. We demonstrate that emergent biosynthetic capacity – the ability of multiple species growing together to produce and secrete metabolites that none of the member species secretes when growing alone – is common, and identify typical reprogramming mechanisms and temporal patterns that underlie this capacity. Importantly, we show that emergent capacity is most likely when the species comprising the community are neither too functionally similar nor too distant. Overall, our findings lay the foundation for a comprehensive and predictive understanding of synergistic microbial activity and highlight promising routes for designing, engineering, and manipulating microbial communities toward desired metabolic capabilities.

## Introduction

Microbes have a remarkable capacity to transform their environments, converting key nutrients and energy into accessible forms that are essential for the survival of all other organisms [1], [2]. This capacity is mediated by a plethora of interactions with the environment and a complex web of metabolic reactions occurring within each microbial cell. In nature, however, most microbes do not typically exist in isolation but rather form complex and diverse communities, joining forces to accomplish tasks that may be energetically unfavorable if performed by a single species [3], [4]. Such communities play a central role in ecosystem dynamics, agriculture, environmental stewardship, and human health [5]–[9]. The various species comprising each community often form tight relationships and metabolic dependencies [10], [11], which can affect the overall stability of the community and its activity. Synergistic relationships endow microbial consortia with enhanced metabolic capacities including nitrification in soil and marine environments [12], methane oxidation [13], and pesticide degradation [14], further affecting the interplay of the community with its environment [15].

Considering these dependencies and synergistic relationships, the metabolic capacity of a given microbial community clearly cannot be described simply as the aggregated capacity of its member species, and a deeper understanding of how the activity of each community member impacts the activity of the others and ultimately the behavior of the community as a whole is required. Of specific interest are cases where the biosynthetic activity of the community is fundamentally different from the biosynthetic activity of the member species. This is often the result of a simple niche construction process [16], wherein the secretion or uptake of metabolites by one species modifies the composition of the environment and consequently modulates the metabolic activity of another species, causing it to produce and secrete metabolites it would not have produced if growing in isolation [3], [17]. Characterizing such phenomena, which we term here *emergent biosynthetic capacity*, is crucial for understanding how microbes jointly construct their environment. More importantly, understanding the determinants of emergent biosynthetic capacity and ultimately designing microbial communities that exhibit specific metabolic capabilities, is a promising research avenue with many industrial and clinical applications, ranging from biofuel production to personalized microbiome-based therapy [3], [17]–[22]. Here, we therefore set out to characterize emergent biosynthetic capacity on a large-scale and to inform future efforts to design microbial communities that perform desired metabolic tasks.

One approach to study the metabolic capacity of microorganisms and to obtain a systems-level predictive understanding of microbial metabolism is through metabolic modeling. Specifically, genome-scale metabolic models have been instrumental in providing insights into the metabolism of various microbial species, their ecology, and their behavior in different settings [23]–[30]. Of the various genome-scale modeling frameworks, constraint-based modeling (CBM) methods, such as Flux Balance Analysis (FBA), are perhaps the best-established and most commonly used methods [31], [32]. Such methods aim to model cellular metabolism as a set of mass balance, thermodynamic, and flux capacity constraints, and to predict the growth rate of the organism as well as the specific distribution of fluxes across the metabolic network by optimizing a cellular objective such as cellular growth or energy production. This modeling framework was shown to correctly capture various factors that govern microbial metabolic processes, providing mechanistic insights into microbial metabolism [28], [32]. Most importantly, such models proved extremely successful in accurately predicting microbial behavior and activity in multiple environments and under various perturbations [33], with numerous clinical, environmental, and industrial applications [34], [35].

With the increased availability of high-quality, manually curated single species models [36] and the recent introduction of automated model reconstruction pipelines [37], [38], constraint-based methods provide a unique opportunity to model multi-species ecosystems and to study their metabolic capacities [39]–[42]. However, integrating multiple single species models and developing a framework for modeling diverse microbial communities is not a simple and straightforward undertaking, and to date relatively few CBM-based multi-species models have been presented. One critical problem is how to properly define an objective function at a community level. Stolyar *et al.* [43] introduced the first two-species FBA model to study a methanogenic syntrophic system, using an objective function that maximizes a fixed combination of biomass from two organisms. A similar approach, maximizing the sum of individual species growth as an overall community objective, has been applied to capture metabolic interactions between ecologically associated species [44], and to predict measured phenotypes of representative gut microbiome species [45]. Notably, however, the overall community growth objective inherently assumes that member species cooperate and act for the common good of the community, which may potentially lead to biased predictions, wherein, for example, one species barely grows (although nutrients are available) to enable the growth of another. One approach to relax this overall community growth objective using a multi-layer optimization algorithm to introduce trade-offs between individual and community level optimization criteria has recently been proposed, and applied to study syntrophic interactions in a few well-characterized, multi-species microbial systems [46]. Alternative optimization methods have also been used to study synthetic cooperation between single gene deletion mutants [47], environments that induce species cooperation [48], and diet dependent changes in uptake and secretions between a host and a dominant gut microbe [49]. Yet, the methods above often assume a predefined community composition, a certain level of optimality for each species, or a well characterized species interaction pattern, and may not be easily generalized to predict the consequences that the introduction of one species may have on the metabolism of another or to systematically study the metabolic capacity of microbial ecosystems.

One fundamentally different approach to tackle this challenge is to incorporate temporal dynamics into these modeling frameworks. Previously, temporal dynamics have been successfully incorporated into single-species models to predict metabolic reprogramming, growth, and secretion rates [50]–[53]. Recently, a few preliminary studies have similarly used this approach to study microbial co-cultures composed of sub-populations of strains or of multiple species [54]–[58]. In several such dynamics-based studies each species aims to maximize its own growth on a short time-scale and the overall community dynamics is a long time-scale integration of species dynamics. This modeling framework is not dependent on maximizing community growth and more importantly, is especially suited for studying the biosynthetic and secretion capacities of microbial communities over time.

Here we introduce a comprehensive computational framework tailored specifically for studying emergent biosynthetic capacity in simple microbial communities. We extend recently introduced dynamical modeling frameworks, presenting a multi-scale model that tracks both the metabolic activity of each species over time and the effect of this activity on the concentration of various metabolites in the environment. This framework therefore allows us to examine how environmental shifts induced by the activity of one species may impact the activity of another. Using this framework to model the growth of both single- and two-species microbial systems, we aim to identify instances wherein a two-species community secretes certain metabolites that cannot be secreted by any of the member species when grown in isolation. Notably, to obtain confident predictions of emergent capacity, our framework further incorporates flux variability-based techniques to account for multiple alternative FBA predictions. We first use a simple toy ecosystem model to demonstrate the ability of our framework to detect emergent biosynthetic capacity. Next, we utilize high-quality, manually curated, and previously validated genome-scale models of six microbial species to systematically characterize emergent biosynthetic capacity across a large array of growth media. We specifically set out to examine how common emergent capacity is, within which growth phase does it most frequently occur, and what combinations of microbial species are most likely to exhibit emergent capacity. We further characterize several typical mechanisms underlying emergent biosynthetic capacity and explore frequent emergent metabolites. Finally, we apply this framework to a large collection of automatically reconstructed models of >100 microbial species to validate the observed patterns on a broader scale. Taken together, our results highlight promising directions for studying unique metabolic capacities of microbial communities, facilitating future efforts to steer complex ecosystems and their environments towards beneficial states.

## Results

### A Framework for Characterizing Emergent Biosynthetic Capacity

**Figure 1:**
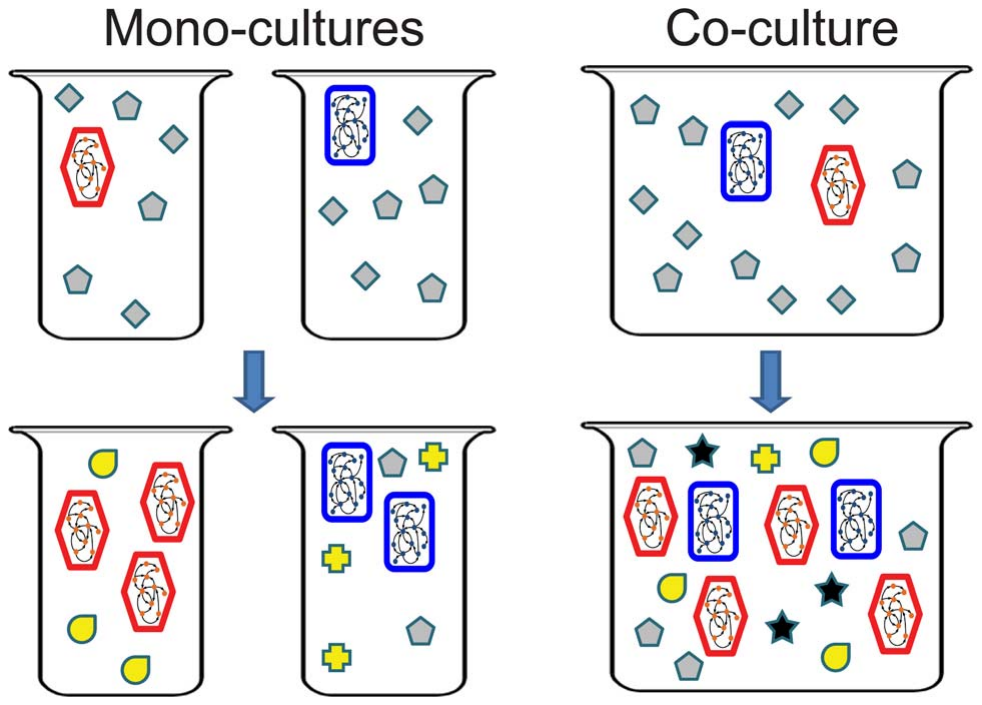
Emergent biosynthetic capacity in a simple microbial community. In this simple illustration, the co-culture secretes several metabolites (e.g., the yellow tear- and cross-shaped metabolites) but only one metabolite (star-shaped) is considered emergent.

To systematically characterize the emergent biosynthetic capacity of microbial communities, we focus here on simple two-species microbial ecosystems. Formally, given two species and a predefined growth medium, we define *emergent metabolites* as metabolites that are secreted and consequently accumulate in the environment when the two species grow in co-culture but that are not secreted by either of the two species when they grow in mono-culture (Figure 1). A two-species system in a given medium is then said to exhibit *emergent biosynthetic capacity* if it secretes at least one emergent metabolite. Notably, this is a very strict definition, as we do not consider the potentially many cases wherein the secretion *rate* of some metabolite is higher in co-culture than in mono-culture, but rather focus on cases wherein the co-culture system secretes metabolites that are completely absent in the two mono-culture systems. This definition allows us to study the prevalence and determinants of fundamentally novel behavior of microbial ecosystems, rather than quantitative and potentially minor differences. Furthermore, as described below, this definition may be less sensitive to parameter selection or other inaccuracies in the underlying model (e.g., in the bounds set for the uptake rate of nutrients). Moreover, we only consider “neutral” growth media that allow each species to grow in mono-culture, rather than media that explicitly induce commensal or mutualistic interactions (and see also ref. [48]). Accordingly, we go beyond studies of species symbiosis (e.g., [59]) and specifically target scenarios where emergent capacity is not simply the outcome of one or both species surviving only due to the association with the other.

Following these definitions, we developed a computational framework for detecting emergent metabolites and emergent biosynthetic capacity. Our framework integrates genome-scale metabolic models, temporal dynamics, ecosystem settings, and various optimization schemes. To simulate the growth of a two-species system in a given growth medium over time and its impact on the medium, we followed previous studies [54], [57], using a multi-scale dynamic FBA-based framework. Rather than defining an arbitrary community objective that governs the flux distribution of the system, this framework assumes that each species in the community seeks maximum growth. Briefly, in this framework, we first predict the behavior of each species (including flux activity and growth rate) in the initial medium within a short time interval, Δ*t*, using FBA [50]. We use Michaelis-Menten equations and scale metabolites by the total cell density and time interval, to estimate nutrient availability and the allocation of nutrients among the species. We then determine the cell density of each species according to the predicted growth rate and update the concentration of metabolites in the medium at the end of this time interval based on the predicted uptake and secretion fluxes of the various species. Notably, as we are using a shared medium, species can then utilize metabolites secreted by other species. We perform these two steps repeatedly, each time using the updated medium composition as the initial medium for the following iteration. Simulations continue until nutrients are exhausted and all species stop growing. Notably, our framework simulates a batch culture condition, following typical dynamic FBA studies [50]–[53]. Throughout the process, we record the concentration of metabolites in the medium, obtaining a full characterization of the co-culture and medium over time. A detailed description of this ecosystem model is provided in the Methods.

To detect emergent metabolites, we simulate the growth of each species in mono-culture in a similar manner, again recording the composition of the medium at each time step. We then mine these records to identify metabolites that occur in the co-culture medium at some point throughout the growth of the species, but that never occur in the medium of either mono-culture. Importantly, FBA provides only a single flux solution, whereas many alternative solutions with equally optimal growth rates may exist. Considering our definition above, we therefore wish to confirm that candidate emergent metabolites are not only absent from the specific solution obtained, but are absent from *any* possible solution (under the optimal growth criterion). Notably, characterizing all possible solutions over time is a challenging task as we need to account not only for alternative solutions in a specific time point but rather for alternative solutions in all time points *and* their potential impact on subsequent time points. The space of alternative solutions may therefore expand exponentially with time as the set of solutions in each time point may depend on the solution employed in previous time points. To address this challenge, we developed an *iterative flux variability analysis* (see Methods) as a more stringent protocol for detecting emergent metabolites that accounts for this potentially expanding set of secreted metabolites. We then classify metabolites as emergent only if they do not appear in any of the alternative solutions obtained by this iterative analysis. We confirmed that this protocol filters out potentially spurious results that rely on assuming specific alternative solutions during the growth period (see Methods). The results presented below are accordingly obtained using this stringent protocol.

As described below, we applied this framework to several sets of two-species ecosystems growing on a large set of neutral media. For single-species models, we used both manually-curated, high quality genome-scale reconstructions and automatically generated reconstructions obtained from previously published studies (Methods). Neutral media were obtained from previous studies or generated through an optimization algorithm (see Methods).

### A Toy Ecosystem with Emergent Biosynthetic Capacity

**Figure 2:**
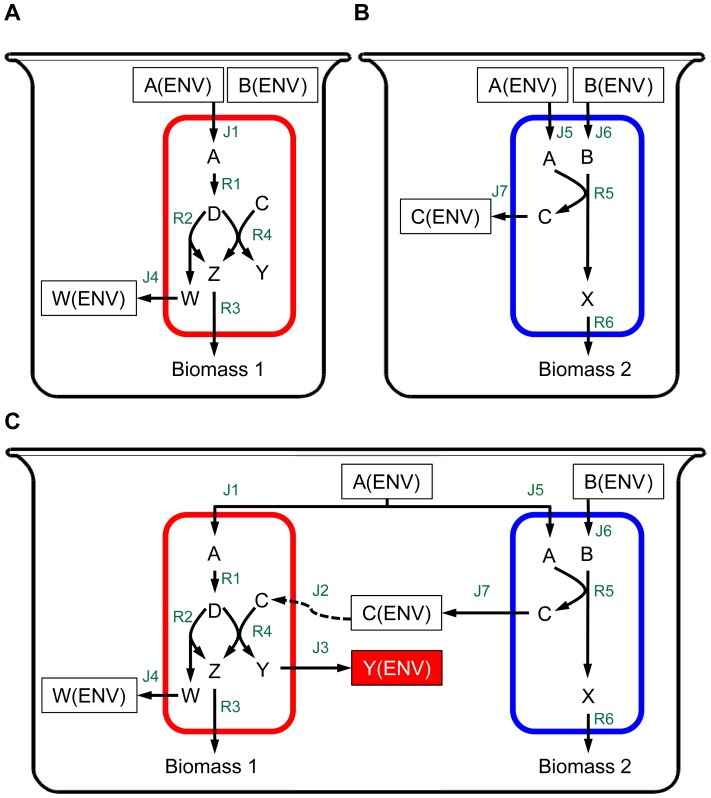
A toy ecosystem of emergent biosynthetic capacity. Both the red (**A**) and blue (**B**) species can successfully grow in mono-culture using the same medium that contains metabolites A and B. Yet, growing the two species in co-culture (**C**), an emergent biosynthetic capacity can be observed, as the red species utilized via cross-feeding (dashed lines) metabolite C that was secreted to the medium by the blue species, activating an alternative pathway for biomass production and secreting metabolite Y as a byproduct.

To illustrate the settings that can give rise to emergent biosynthetic capacity and the ability of our computational framework to detect it, we first present a simple toy ecosystem in which an environmental shift induced by one species promotes a second species to activate an alternative pathway and consequently secrete an emergent metabolite. Specifically, consider the two species illustrated in Figure 2A–B (and see Methods for a full model description). Each of these species can successfully grow in mono-culture on the same simple medium (containing nutrients A and B). In the process of converting exogenously acquired nutrients to biomass, the red species secretes metabolite W to the medium as a byproduct (Figure 2A), while the blue species secretes metabolite C (Figure 2B). However, when grown in co-culture (and on the same medium as in mono-culture), the red species has access to metabolite C that was secreted by the blue species (Figure 2C, dashed arrow), allowing it to utilize an alternative pathway for synthesizing metabolite Z – a precursor of biomass. If this alternative pathway is favorable for optimal growth (see, for example, the stoichiometric details of this model in Methods), the red species may activate this pathway to produce more biomass and grow faster, secreting metabolite Y as a byproduct in the process. Since this metabolite Y cannot be secreted by either of the mono-cultures, it is classified as an emergent metabolite and this system is classified as exhibiting emergent biosynthetic capacity. Notably, the definition of emergent biosynthetic capacity is medium-dependent; for example, in this toy ecosystem, Y will not be classified as an emergent metabolite if the growth medium already contains metabolite C since in such a scenario, metabolite Y can be secreted also by the red species in mono-culture.

**Figure 3:**
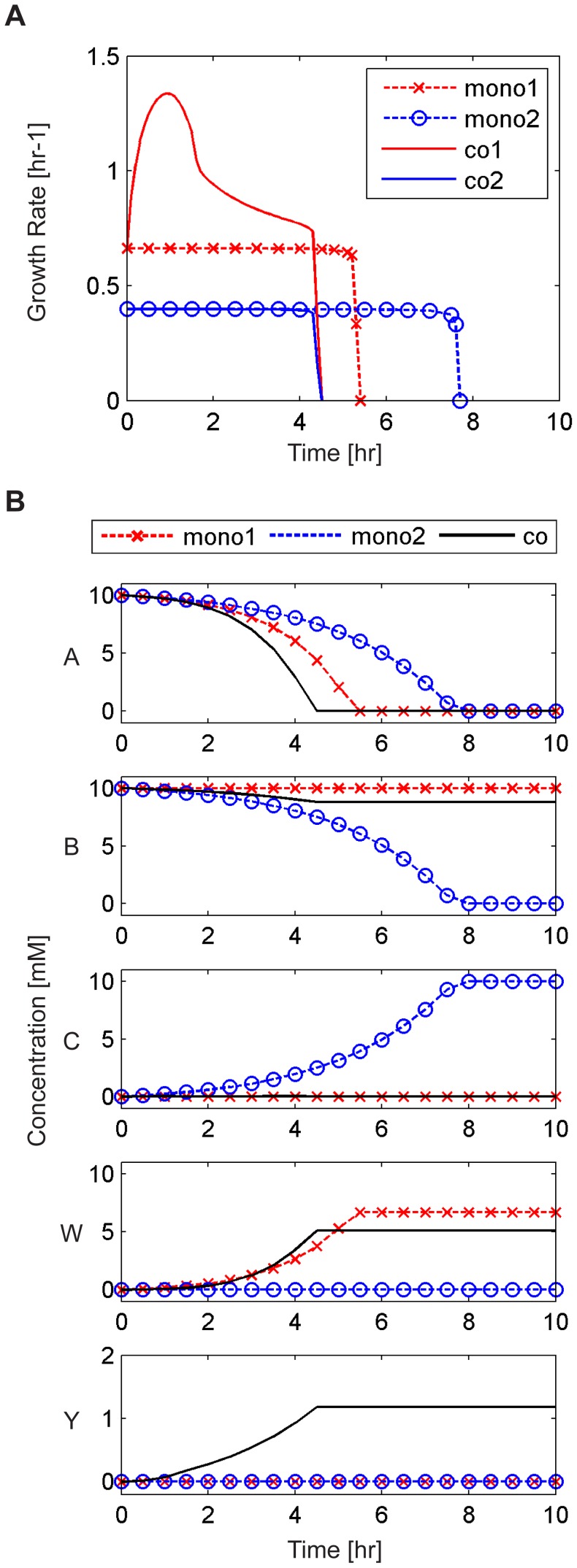
Temporal dynamics obtained by our framework for the mono-cultures and co-culture of the toy ecosystem illustrated in Figure 2. (**A**) Growth rate of the red and blue species in mono-cultures and co-culture. (**B**) The concentration of various metabolites in the medium over time in the mono-cultures and co-culture. Metabolite Y was identified as an emergent metabolite since it was secreted in co-culture (solid line) but was totally absent from either of the two mono-cultures (dashed lines). In contrast, metabolites W and C were observed in the medium in at least one of the mono-cultures (although at different concentrations than in co-culture).

Applying our framework to this ecosystem, this emergent capacity was clearly observed (Figure 3). Evidently, the availability of metabolite C in co-culture allowed the red species to grow faster than it grew in mono-culture (Figure 3A). Tracking the concentration of various metabolites in co-culture and in mono-cultures (Figure 3B), several differences were further observed. For example, the improved growth of the red species in co-culture led to a faster depletion of metabolite A, which was accordingly exhausted earlier, preventing further growth. Most notably, however, metabolite Y (Figure 3B, lower panel), which was completely absent in either of the mono-cultures, quickly accumulated in co-culture, owing to the activation of the alternative pathway in the red species. Comparing the concentration of metabolites in co-culture with those obtained in mono-culture and applying our variability analysis to examine alternative solutions (Methods), our framework therefore identified Y as an emergent metabolite.

### Characterizing the Prevalence of Emergent Biosynthetic Capacity and of Emergent Metabolites

The toy ecosystem above was specifically designed to promote emergent biosynthetic capacity. While such synergistic capacities can clearly occur in communities of real microorganisms, it is not clear *a priori* whether it is common, how likely it is to occur, and which factors may contribute to it. Next, we therefore set out to examine how prevalent emergent biosynthetic capacity is in natural systems and what the determinants of such capacity are in simple microbial communities. To this end we obtained high-quality genome-scale metabolic models of 6 species with potential health and environmental applications (Methods). Importantly, each of these models was manually curated, experimentally tested, and used in multiple previous studies of microbial metabolism [48]. We then applied our framework to characterize emergent biosynthetic capacity in all possible pairwise species communities. Since, as discussed above, emergent capacity is media-dependent, we simulated the growth of each of these two-species communities in 100 random minimal neutral media (see Methods) and recorded the cases in which emergent capacity occurred.

**Figure 4:**
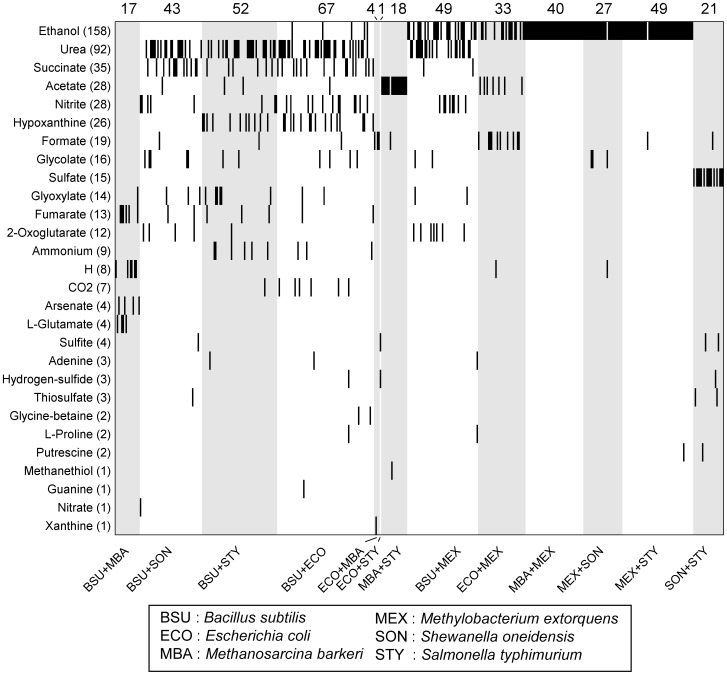
Emergent metabolites in simple two-species communities. Rows represent emergent metabolites and are ranked by their prevalence. Each column represents a specific two-species community growing in a random neutral medium. Secretion of a specific emergent metabolite in a certain community/medium combination is illustrated with a black bar. Only the 421 community/medium combinations that exhibited emergent biosynthetic capacity (*i.e.*, at least one emergent metabolite) are shown. The species comprising each community are shown on the bottom, and the number of media in which emergent biosynthetic capacity occurred for each of these communities is shown on the top.

Interestingly, our analysis demonstrated that emergent biosynthetic capacity is fairly common. Almost all pairwise species combinations analyzed (13 out of 14) exhibited emergent capacity in at least one of the 100 media, with some species pairs (e.g., *E. coli* and *B. subtilis*) exhibiting emergent biosynthetic capacity in 67 of the 100 tested media (Figure 4). Overall, 30% (421) of the 1400 community/medium settings analyzed demonstrated emergent capacity. Notably, in most cases (343 of 421) communities with emergent capacity secreted only 1 emergent metabolite, but in a few cases (12 of 421) 3 different emergent metabolites were secreted simultaneously. Interestingly, in 16 community/medium settings (mostly involving *E. coli* and *B. subtilis*), both species secreted emergent metabolites that were consequently consumed by the other species via cross-feeding, exhibiting an intriguing mutual emergence scenario.

Focusing on the emergent metabolites that were identified in each simulation, we found in total 28 metabolites that were classified as emergent in at least one community/medium. Of these, ethanol and urea were the most frequently secreted emergent metabolites (37.5% and 21.9% of the cases in which emergent biosynthetic capacity was detected, respectively). Figure 4 lists the complete set of emergent metabolites, ranked by their frequency, and the communities in which they were detected. Perhaps not surprisingly, the most frequently secreted emergent metabolites include common byproducts of microbial metabolism, such as ethanol, succinate, and acetate (from fermentation), and urea (from nitrogen metabolism). Several of these metabolites (e.g. ethanol, acetate, urea, glycolate) have been previously highlighted as important cross-feeding metabolites that can substantially impact the composition of various microbial ecosystems [60]–[62].

To better understand the contribution of each species in the community to the observed emergent biosynthetic capacity, we further examined each community with an emergent metabolite, to determine which of the two species actually secreted that metabolite to the environment (the *producer*) and which species was the non-secreting community member (the *partner*). Focusing on the most frequently secreted emergent metabolites, we found that emergent metabolites were often secreted by a single dominant producer species, whereas many other species could serve as partners (Figure S1). Critically, many dominant producer species have been shown experimentally to secrete the compound in question given the proper environmental conditions and media. For example, succinate secretion has been demonstrated in *B. subtilis* [63] and *E. coli* [64]. Similarly, the primary producers of acetate in our study, *S. typhimurium*, *M. barkeri*, and *E. coli*, are capable of secreting appreciable quantities of this compound under certain conditions [65]–[67]. This analysis further demonstrated that *B. subtilis* was almost solely responsible for the secretion of several frequent emergent metabolites, including urea, nitrite, glyoxylate, and fumarate, partnering with almost any other species. Notably, *B. subtilis* was the only species included in our analysis that has a complete urea cycle, providing metabolic flexibility for nitrogen and amino acid metabolism and potential utilization of varied nutrient sources. Indeed, some of the emergent metabolites secreted by *B. subtilis* are associated with the operation of the urea cycle (see also Figure S3) and have been shown to be the end product of this process in other microorganisms with a complete urea cycle [68]. This promiscuity of *B. subtilis* may also reflect its adaption to diverse habitats including soil, plant roots, aquatic environments, and the gastrointestinal tract of animals [69]. In other cases, however, only a very specific combination (or combinations) of species led to the secretion of an emergent metabolite (e.g., Sulfate; Figure S1). Interestingly, comparing the total biomass produced in co-culture to the combined biomass produced by the two mono-cultures, we found that most (but not all) communities benefit from such emergent capacity, even though overall community growth was not the optimization objective in our framework (see Text S1 and Figure S8).

### Mechanisms of Emergent Biosynthetic Capacity

The two species comprising the toy ecosystem discussed above demonstrate a simple mechanism of emergent capacity. Specifically, in this example, one organism constructed its niche, converting nutrients or energy sources (metabolites A and B) into forms accessible to other species (metabolite C). Species that share this niche may consequently “reprogram” their metabolic activity (e.g., via regulation) to obtain optimal growth and differentially impact their environment (e.g., by secreting metabolite Y). Clearly, however, in real organisms, which can catalyze hundreds and thousands of reactions, metabolic reprogramming can be markedly more complex, involving differential activation of numerous reactions in response to environmental shifts. Such reprogramming may, for example, activate (or enhance) some reactions while deactivating (or repressing) other reactions, to support multiple nutrient requirements, energy production, and redox balance. Here, we therefore set out to examine whether similar cross feeding-based mechanisms were responsible for the prevalence of emergent capacity observed above in models of real microbial species and to characterize metabolic reprogramming patterns that may be associated with this capacity.

We analyzed the metabolic fluxes in each community that exhibited emergent biosynthetic capacity, comparing the predicted fluxes in co-culture with those predicted in the two mono-cultures. We identified in each two species system the species that secreted the emergent metabolite (*i.e.*, the producer; and see also Figure S1) and the time point at which the emergent metabolite was first secreted. To examine whether a simple cross-feeding behavior could account for the observed emergent capacity, we first aimed to identify cross-feeding fluxes at this time point that might have prompted the producer to secrete the emergent metabolite. Specifically, we identified metabolites taken up by the producer that were not provided in the initial growth medium and that were not secreted by the producer itself in earlier time points. To confirm that these cross-feeding fluxes were sufficient to induce the secretion of an emergent metabolite, we simulated the growth of the producer in mono-culture again, with small amounts of the detected cross-feeding metabolites added to the medium. We found that in almost all cases (99.4%) the addition of these metabolites indeed resulted in a reprogramming event, altering the activity of the producer and causing it to secrete the emergent metabolite. The few cases (3) where this did not occur may require the presence of additional emergent metabolites that were secreted by the producer at earlier time points (and that were therefore not added to the medium) or the availability of the cross-feeding metabolite at a higher concentration. Further analysis also demonstrated that in most cases (∼98%) emergent metabolites could in fact be secreted by the producer also in mono-culture but that such secretions were suppressed when biomass production was maximized (Text S1 and Figure S9). As this growth penalty was often relatively small, it appears that the role of the partner in many communities amounted to reducing the cost of specific emergent secretions by allowing the producer to shift its metabolic activity toward a pattern that was suboptimal when growing in isolation, and only in some cases did the partner actually provide metabolic capabilities necessary for emergent secretion.

**Figure 5:**
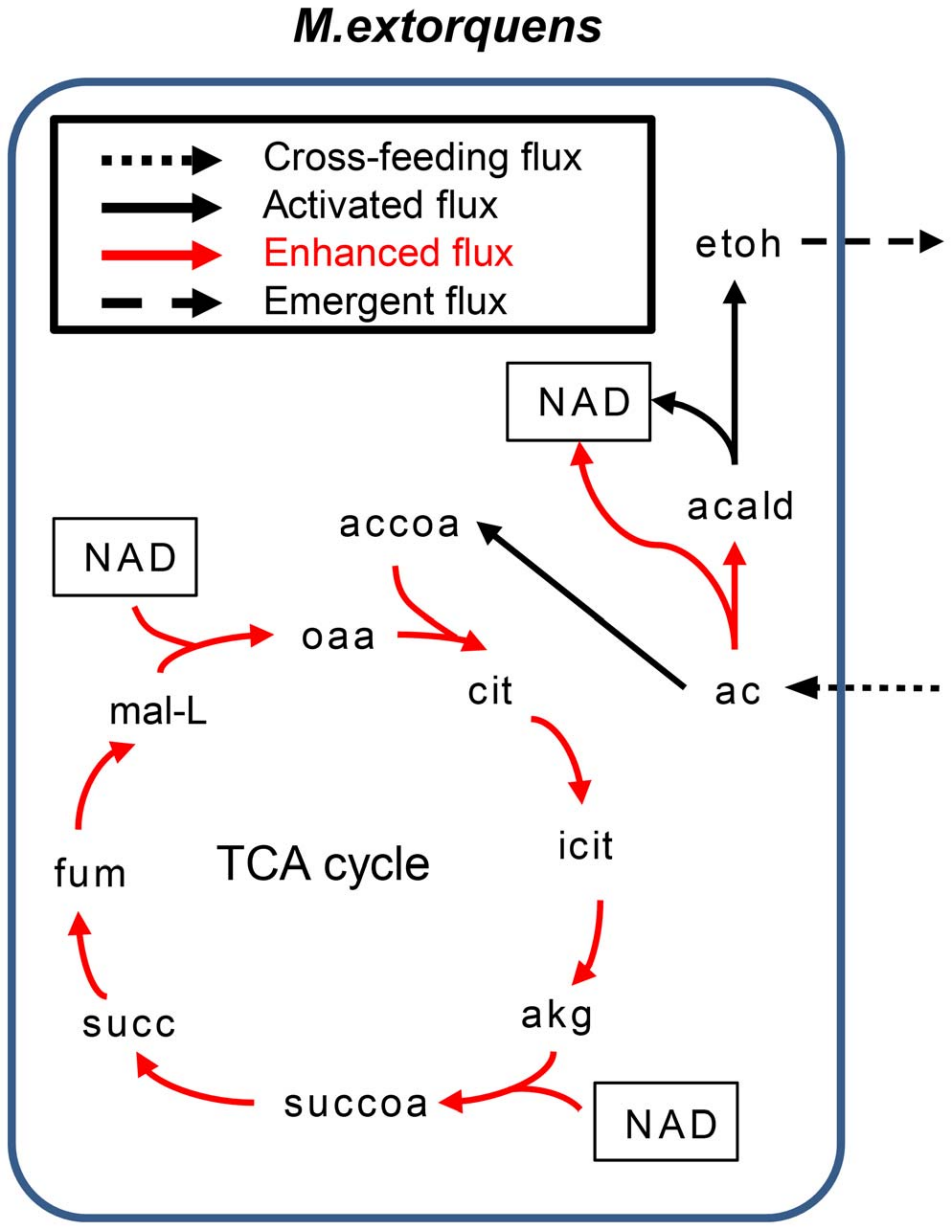
Metabolic reprogramming of *M. extorquens* that results in ethanol secretion. Major reprogrammed fluxes (compared to the mono-culture settings) are plotted. In this example, acetate that was secreted to the medium by *S. oneidensis*, was taken up by *M. extorquens* and converted into acetylCoA, leading to increased fluxes in the TCA cycle. A small portion of the acquired acetate was converted into ethanol through acetaldehyde to replenish NAD^+^ and to facilitate energy production by the TCA cycle. Abbreviations - etoh: ethanol; acald: acetaldehyde; ac: acetate; accoa: acetyl-CoA; cit: citrate; icit: Isocitrate; akg: 2-oxoglutarate; succoa: succinyl-CoA; succ: succinate; fum: fumarate; mal-L: L-malate; oaa: oxaloacetate; NAD: nicotinamide adenine dinucleotide.

Clearly, one of the benefits of a modeling framework is that it allows us to comprehensively characterize flux distributions in each organism and to fully characterize complex reprogramming behaviors. Since ethanol and urea were the two most frequently secreted emergent metabolites observed in our analysis, we examined several common cases of metabolic reprogramming that resulted in the secretion of these metabolites in more detail and illustrated two typical examples of such reprogramming patterns. In the first example, acetate was identified as a cross-feeding metabolite from *Shewanella oneidensis* to *Methylobacterium extorquens*, resulting in emergent ethanol production (Figure 5). Specifically, the uptake of acetate allowed *M. extorquens* to enhance energy production by providing increased carbon flow into the TCA cycle. Acetate influx additionally enhanced acetyldehyde dehydrogenase and activated alcohol dehydrogenase fluxes through which NAD^+^ was generated. This excess NAD^+^ production allowed reactions catalyzed by malate dehydrogenase and 2-oxoglutarate dehydrogenase to regenerate NADH in the TCA cycle as a source of ATP as well as reducing power that were necessary in many other reactions. Notably, a similar phenomenon, wherein exogenous acetate induces ethanol secretion to maintain intracellular redox balance, has been previously documented in *Lactobacillus casei* [70]. Emergent secretion of ethanol to maintain redox balance was also observed in our analysis in many other communities and in various growth media, often involving markedly more complex reprogramming patterns. For example, in the case illustrated in Figure S2, ethanol was produced as an emergent metabolite by *E. coli* in the presence of *B. subtilis* following a complex reprogramming behavior that involved not only enhancement and activation of various reactions but also flux repression in several branch points to adjust carbon flow into the TCA cycle. Furthermore, the optimization of NADH production appeared to be a common strategy that resulted in emergent ethanol secretion.

As a second example we focused on a community that secreted urea and succinate by *B. subtilis* and *E. coli* respectively (Figure S3). In this example, the metabolite fumarate cross-fed from *B. subtilis* to *E. coli* and promoted energy production as well as amino acid biosynthesis in *E. coli*. Fumarate uptake by *E. coli* was partly mediated by a dicarboxylic acid transporter that resulted in succinate secretion. Utilization of a dicarboxylic acid transporter for fumarate uptake that is accompanied by succinate secretion has been previously documented in *E. coli* in certain media [71]. Meanwhile, acetate cross-feeding from *E. coli* to *B. subtilis* allowed a larger carbon flow into the TCA cycle in *B. subtilis*, resulting not only in more energy but also in more 2-oxaloglutarate production by *B. subtilis*. Consequently, the amount of glutamate required to generate 2-oxaloglutarate and NDPH was decreased (Glutamate:NADP oxidoreductase), and excess glutamate was rerouted to the urea cycle to facilitate arginine biosynthesis. Arginase was activated in this new metabolic route, ultimately resulting in the secretion of urea.

### Early and Late Onset of Emergent Capacity

In determining the prevalence of emergent capacity above and in characterizing various underlying mechanisms of this capacity, we compared the activity of the co-culture and mono-cultures during the initial phase of growth. Specifically, for the results reported in the previous sections we focused on all time points up to the 1 hr time point. During this period organisms in our simulations were still exhibiting exponential growth and were not limited by nutrient availability. However, as cell density increases and nutrients get depleted, organisms may further modulate their activity to optimize their growth in a nutrient limited environment. This may potentially lead to different modes of emergent capacity and to the secretion of emergent metabolites that may not be observed during early growth.

**Figure 6:**
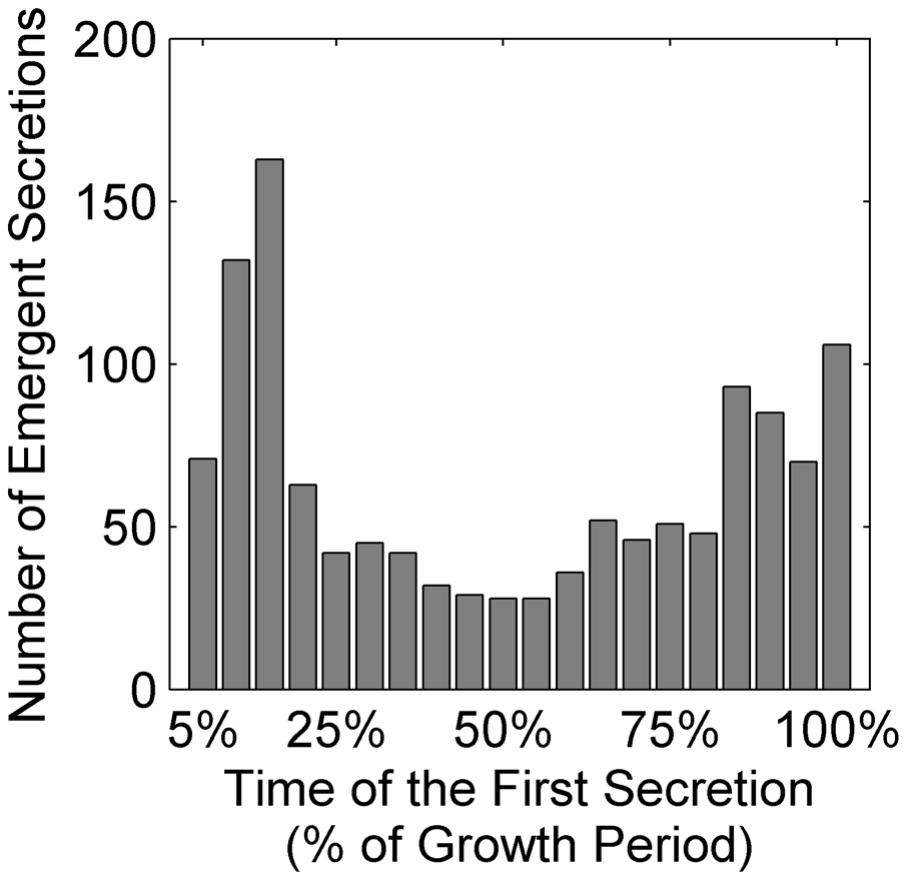
The number of emergent secretion events detected throughout the growth of all community/media combinations included in our analysis. The earliest secretion time point for each event was recorded and presented here as the percentage of the entire growth period in the corresponding community/media settings to account for variability in total growth time among the different simulations.

To test this hypothesis, we repeated the analysis above considering the entire growth period (*i.e.*, until some required nutrients were totally exhausted and growth ceases) and for each emergent biosynthetic event recorded the first time point the emergent metabolite was secreted. Examining the distribution of such events over time, we indeed found two waves of emergent capacity, one that occurred mostly during the early growth period, and one that occurred toward the end of the growth period (Figure 6). While the first wave represents emergent capacity that arose as soon as the two species were introduced into the same medium, the second wave may reflect emergent capacity that was dependent upon organisms' activity in a nutrient depleted media. In total, when the entire growth period was considered, 52% (729) of the 1400 community/medium settings analyzed demonstrated emergent capacity (compared to the 30% reported above when only the early growth period was considered). During this full period, all 14 pairwise species combinations analyzed exhibited emergent capacity in at least several different media, with some species combinations exhibiting such capacity in >80 of the 100 media tested (see Figure S4). Again, in most cases (414 of 729) only a single emergent metabolite was secreted, but in one case as many as 11 emergent metabolites were secreted in the same community/medium combination.

To further characterize the differences and similarities between these two waves of emergent capacity, we compared the set of emergent metabolites reported above for the initial growth period (1 hr) to the larger set of emergent metabolites obtained when the entire growth period was considered. As expected, this latter set was markedly larger, with 64 different metabolites (compared to the 28 identified in the 1 hr set) classified as emergent in at least one community/medium (Figure S4). Specifically, among the metabolites that were detected as emergent only in the late growth period, glycolaldehyde, malate, and propionate were the most prevalent. Interestingly, propionate was previously observed as a late onset emergent metabolite in a co-culture of *Megasphaera elsdenii* and *Streptococcus bovis* [72]. In that system, propionate was produced by *M. elsdenii* as a result of secondary fermentation of lactate that was secreted to the medium by *S. bovis* and was detected only 3 hours after the simultaneous inoculation of both species once lactate accumulation was sufficient [72]. Other emergent metabolites that were detected only in late growth may reflect similar processes, wherein one species shifted to metabolize secondary byproducts secreted by the partner. In other cases, however, late detection of emergent metabolites may depend on specific thresholds used. For example, glycolaldehyde, which was produced as an emergent metabolite by *M. barkeri*, was not detected in the early growth period due to the slow growth of this species and consequently the long time required for glycolaldehyde to accumulate in the medium and to pass the threshold used. More generally, however, while certain emergent events could only be observed in late growth as described above, the results obtained for the 1 hr time point and those obtained for the entire growth period were consistent (see also Figure S5), with ethanol, succinate, and urea being the most frequently secreted emergent metabolites.

### Community Compositions Likely to Exhibit Emergent Capacity: The Goldilocks Principle

Above, we demonstrated that emergent biosynthetic capacity is generally prevalent in simple two-species communities. Here, we set out to detect properties of the community that may be associated with this capacity and that can help us to determine *a-priori* which community compositions are more likely to exhibit emergent capacity. Identifying such properties can inform future design efforts, allowing them to focus the search for novel metabolic activity on specific species combinations. We specifically examined whether the functional and phylogenetic distance between community members was a potential determinant of emergent biosynthetic capacity and of the ability of two species to interact and exhibit a novel behavior.

**Figure 7:**
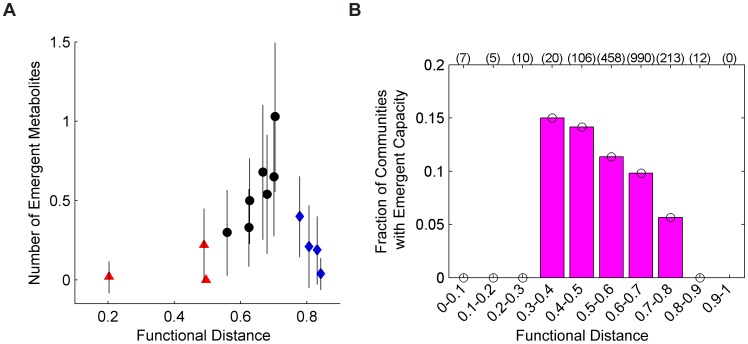
The Goldilocks principle of emergent biosynthetic capacity observed in simple two-species communities. (**A**) The average number of emergent metabolites (and standard error) across 100 media in each of the communities containing 2 of the 6 species in our first dataset vs. the functional distance between the two species. Red triangles denote pair-wise combinations of the three proteobacteria *E. coli*, *S. typhimurium*, and *S. oneidensis*. Blue diamonds denote communities composed of a bacterium paired with the archaeon *M. barkeri*. The pair *M. barkeri*/*S. oneidensis* was not included in this analysis since no neutral media for this pair was found in [48]. Functional distances were calculated as the Jaccard distance between the set of KEGG orthology groups [101] present in the two species (Methods). (**B**) The fraction of communities that exhibit emergent biosynthetic capacity across communities containing 2 of the 116 species in our second dataset within a given range of functional distances (Methods). Only the 1821 communities in which both species were still growing at the 1 hr time points are included, with the number of such communities in each bin shown in parentheses.

To this end, we compared the average number of emergent metabolites secreted by each two-species community across random neutral media to the functional and phylogenetic distance between the two species (Methods). As demonstrated in Figure 7A, we found that communities which consisted of functionally close species tended to exhibit low levels of emergent capacity. For example, communities where both species were gamma-proteobacteria (red triangles in Figure 7A) produced on average only 0.08 emergent metabolites. Interestingly, however, communities in which the two species were functionally distant from one another similarly tended to secrete only few emergent metabolites. Specifically, in our dataset, communities containing a bacterium paired with the archaea *M. barkeri* (blue diamonds in Figure 7A) produced on average only 0.21 emergent metabolites. It was only when the two species that comprised the community were at some intermediate functional distance that higher levels of emergent biosynthetic capacity were observed (black dots in Figure 7A). Specifically, the numbers of emergent metabolites in this intermediate group were significantly higher than those observed in both the functionally close species group and the functionally distant species group (*p<10^−18^*; two sample t-test). This “Goldilocks” principle of emergent biosynthetic capacity, that emergent capacity of a community is maximized when member species are not too functionally close, nor too functionally distant, may reflect the ability of species to metabolically interact with one another and beneficially exchange metabolites. Specifically, as discussed further below, functionally similar species may exhibit very comparable metabolic strategies in any given environment, such that the by-products of one species do not provide any novel benefit to the second. In contrast, two functionally distant species may apply markedly different metabolic strategies, such that the metabolites secreted by one species are not compatible with the enzymatic capacity of, and therefore cannot be utilized by, the second species. A similar relationship between distance and the likelihood of emergent capacity could also be observed when using phylogenetic distance rather than functional distance to measure the similarity between the two species (see Figure S6A). Finally, to disentangle the role of species composition from that of the growth media, we repeated the above analysis using a set of 500 ‘universally neutral’ media for all 6 species (Text S1). This analysis further confirmed that the observed Goldilocks principle stems from the functional distance between the two species and is not an artifact of the specific media used for each species pair (Text S1 and Figure S10).

The analysis above, associating optimum emergent capacity levels with an intermediate functional distance between the community members, was demonstrated with high-quality, manually curated metabolic models, but relied on a limited number of species combinations. To confirm the general applicability of this Goldilocks principle, we applied our framework to a markedly larger collection of automatically generated models. These models are potentially less accurate than the manually curated models used above, but represent a significantly higher species diversity and a wider range of functional distances. Specifically, we obtained 116 SEED-based FBA models used in [44], allowing us to examine 6670 two-species communities. Since this dataset did not include a collection of minimal neutral media for each two-species community (as those that were available for the 6 species dataset from [48]), minimal neutral media were computed using a mixed-integer linear programming algorithm (see Methods). Moreover, as simulating the growth of each of the 6670 communities on 100 different growth media is too computationally expensive (Methods), we investigated the level of emergent capacity for each community in one minimal medium (computed specifically for this community) and binned communities into 10 groups representing species pairs with similar functional distances. We recorded the fraction of communities in each bin that exhibited metabolic capacity. Our results mirrored and confirmed those observed in the smaller 6 species dataset (though a significance analysis is challenging with just one medium per community), with an optimum level of emergent capacity obtained by communities in which the two species were at some intermediate functional distance (Figure 7B). Again, a similar pattern held when phylogenetic distance rather than functional distance was used (Figure S6B).

## Discussion

Above we introduced a comprehensive computational framework for exploring enhanced metabolic capacities in simple two-species microbial systems. Investigating a large number of communities and growth media, our results suggest that emergent biosynthetic capacity is relatively prevalent, and can be observed in many, if not all, communities, under certain environmental settings. Notably, while many community/medium combinations exhibited emergent capacities, typically our analysis detected only very few emergent metabolites in each such combination. This may be partly due to our stringent flux variability-based analysis (Methods) that likely filters potentially real emergent metabolites, and thus underestimates the level of species-species interaction and emergent metabolic capacity in nature. Another important factor in determining the prevalence of such capacity is our focus on minimal media that probably provide relatively low nutrient diversity compared to more complex media commonly found in natural habitats. Such naturally-occurring complex environments may allow organisms to utilize multiple resources and potentially produce additional byproducts. Importantly, however, such complex, non-minimal environments may instead allow cohabiting species to partition available resources [73], reducing the impact that one species may have on the other, and consequently the prevalence of emergent biosynthetic capacity. Moreover, as discussed above, our work considered only neutral media, in which both species could survive in isolation. Natural ecosystems, in contrast, often exhibit high levels of *obligate* symbiosis, wherein one species (or both) requires the presence of the other to grow. Such non-neutral media, by definition, promote emergent metabolism, as the activity of the co-culture (in which both species grow) is fundamentally different from that of the two mono-cultures (in which one or both species are not growing). While such scenarios are clearly interesting, here we focused on minimal and neutral media (in which emergent capacity is not a direct outcome of obligate symbiosis) to systematically and comprehensively characterize the limits of emergent biosynthetic capacities without the confounding effects of niche partitioning or obligate symbiosis, laying the foundation for investigating how species jointly influence their shared environment.

Interestingly, our analysis further suggests that emergent biosynthetic capacity is especially likely when community members are neither too similar functionally and phylogenetically, nor too different. This finding is perhaps not surprising: When cohabiting species are too similar functionally, each species is not likely to introduce any metabolite into the environment that the second species cannot already produce by itself, and consequently, the activity of the first species is not likely to modulate the activity of the second. Similarly, if the two species are functionally very different, each species may produce many metabolites that the second species is not capable of producing, but these may be too remote from the metabolism of the second species for it to utilize them. This Goldilocks principle is also in agreement with observations obtained through a simple network expansion analysis [74] or a pathway overlap calculation [75], examining the overall potential of combined metabolic networks. By further characterizing frequently secreted emergent metabolites and examining typical mechanisms of emergent capacity, we were also able to obtain insights into various principles that govern such species-species and species-environment interactions and to point to many potential modes by which microbial species jointly impact their environments. Taken together, our results provide a first systems-level characterization of emergent biosynthetic capacities across simple microbial communities.

As described in the Introduction, several preliminary attempts to model multi-species systems have been previously introduced, some of which have utilized constraint-based modeling approaches or relied on the integration of multiple single-species FBA models (e.g., [44]–[48]). Importantly, the main aim of our study was not necessarily the development of a new modeling framework, but rather the investigation of emergent biosynthetic capacity in microbial communities. Many elements in our framework were therefore adopted from previous studies or adapted from advanced single-species modeling techniques. Yet, as a whole, our framework includes several key innovations that make it especially fitting for studying emergent capacity or, more generally, ecosystems' metabolic activity. For example, similar to Zhuang *et al.* [57], our framework takes a dynamics-based modeling approach. However, Zhuang *et al.* focused on recapitulating a specific, well-characterized community in specific environmental settings, using measured parameters and a species-specific cell death rate. Here, in contrast, we wish to characterize universal principles and large-scale trends of emergent capacity, and therefore focus on a systematic investigation of numerous communities and a plethora of media, for which such detailed information is clearly not available. Our framework, therefore, has integrated an exhaustive Flux Variability Analysis with an iterative temporal modeling approach to account for the potentially many alternative solutions such uncharacterized communities may exhibit. This integration of two constraint-based techniques (namely, Flux Variability Analysis and dynamic-FBA) is essential for a reliable detection of emergent capacity and has not been implemented before.

It is also worthwhile to emphasize again the importance of a dynamics-based approach for studying and discovering emergent community metabolism, since to date, only very few studies have employed this technique whereas most other FBA-based models of multi-species systems have applied a non-dynamic, joint-model approach (e.g., [76]). In such joint-model methods, the stoichiometric matrices of the various species are combined into a single matrix, often with the introduction of explicit exchange reactions. Indeed, such methods are computationally less expensive as they rely on a single (or only a handful of) optimization task(s), without an iterative optimization procedure for tracking the system's dynamics. Yet, in contrast to our method (which optimizes each species' model separately), the size of the stoichiometric joint matrix grows with the number of species, potentially exceeding the capacity of available solvers. More importantly, such methods often require the definition of a universal objective function to represent the optimization objective of the community, such as the total growth of the community or some other community-level feature. Notably, however, a community-level objective may unjustly promote species cooperation, potentially pushing species toward altruistic behavior that could benefit the community. This may be an appropriate approach in cases where cooperation is expected and well-characterized, such as in stable communities of obligate symbiotic pairs (e.g., [76]), but may not be suitable for more general settings in which cooperation is not expected *a priori*. In our framework we therefore instead assume that each species aims to maximize its own growth without regarding the benefit of the community and accordingly optimize each model (representing each species) separately. With this assumption, species impact other community members only by modifying the shared environment as part of this selfish growth process, and a temporal dynamics approach is used to allow environmental shifts induced by the activity of one species to potentially affect the behavior of other species in *subsequent* time points. Moreover, in the context of our study, this dynamics-based approach further allowed us to obtain insights crucial for understanding emergent capacity. For example, cross-feeding metabolites may be secreted at a slow rate, and a long period may be required for such metabolites to accumulate in the environment and reach high enough levels to affect the activity of other species and to induce emergent secretion. Similarly, nutrient availability in the environment can drop over time, pushing community members to shift their flux activity and secrete emergent metabolites only when cell density becomes high. Emergent events can therefore occur at many different time points and may involve different mechanisms, as also demonstrated by our findings on early and late onset of emergent metabolism. Such temporal patterns can only be characterized and investigated with a dynamics-based simulation. More generally, considering the dynamic nature of many natural environments and our focus on emergent secretion events that may further shift the composition of the environment, a dynamical modeling approach capable of tracking these environmental shifts and their temporal impact on the species inhabiting the environment is a natural choice. Ultimately, however, the various approaches for studying community metabolism and the multiple frameworks developed to date are all essential for gaining a comprehensive, principled understanding of microbial communities and of species' metabolic interactions. Future efforts to integrate such different modeling frameworks and to develop a multi-scale framework capable of capturing the many facets of ecosystem metabolism could be especially exciting.

Our modeling framework may clearly have some important limitations. To avoid an arbitrary definition of community objective, we used dynamic FBA-based methods to model community metabolism, assuming that each species aims to maximize its growth and that community dynamics is a second order consequence of species behavior. However, in many natural ecosystems, some species may exhibit sub-optimal growth due to constraints that are currently beyond the scope of FBA. Sub-optimal growth can be observed, for example, when an organism is introduced to a non-natural habitat that it may not have encountered during its evolution. One way to address this challenge is to incorporate additional omic data to augment an FBA-based framework and to guide FBA prediction [53], [77]–[79]. With recent advances in high throughput meta-omic technologies [80], ecosystem-level meta-transcriptomic and meta-proteomic data are continually becoming available and efforts are needed to develop computational methods for incorporating such large-scale meta-omic data into a community-level FBA framework. Other factors that may lead to sub-optimal growth include inhibitory mechanisms such as the bacterial toxin-antitoxin system [81] and inter-species communication mechanisms such as quorum sensing [82]. Such mechanisms are currently not accounted for by genome scale metabolic models. More generally, although efforts have been made to take into account potential sub-optimal growth of community members [46], the rationale and the extent of sub-optimality remains unclear. Other factors may further impact community growth and species interaction. For example, some organisms may have adapted to harsh habitats, such as high temperature or salt concentrations that are stressful to other organisms. Spatial structure could also constrain inter-species metabolic flow and has been shown to induce species cooperation within a remarkably short time of laboratory evolution [83]. Integrating such physical-chemical factors into an enzymatic- or stoichiometric-base framework is a challenging task and will require further developments. Recently introduced efforts to model multiple cellular processes on a whole cell level [84] or to mathematically model spatial constraints among interacting partners [85] are promising advances toward this goal. Ultimately, however, any computational or mathematical model aiming to capture the activity and dynamics of microbial communities or their impact on the environment is bound to be incomplete and may fail to incorporate various factors that could affect the behavior of the community. Environmental attributes, the induction of stress, pH, alcohol, antibiotics, and signaling may all steer the behavior of a specific community away from our metabolic model-based prediction. In this study we therefore aimed to identify large-scale patterns of emergent capacity and to generate hypotheses concerning the universal principles that govern emergent behavior, rather than to predict the metabolic activity of a specific species pair in a specific medium. These robust large-scale patterns (such as the prevalence of emergent capacity or the Goldilocks principle) are potentially less sensitive to model incompleteness and allow us to obtain fundamental insights concerning the capacity, timing, and likelihood of emergent biosynthesis. In this context, a metabolic modeling-based framework further provides a tool for studying an ideal, well-controlled *metabolic* system, ignoring other confounding processes and focusing on mapping of boundaries and first principles of emergent behavior that is governed by metabolism alone.

One of the promises underlying microbial ecology research is the potential for therapeutic or bioengineering applications [41]. In contrast to traditional efforts aimed to genetically engineer a single species toward desired metabolic tasks, engineering microbial communities by constructing specialized combinations of already existing strains is a cost-effective solution [3]. Many clinical and industrial applications may be difficult to address at the single species level but could be potentially attainable at the community level. Of specific interest are microbiome-based therapy applications and gut microbiome transplantation efforts aiming to restore healthy phenotypes or endow the host with some metabolic capabilities [20]. Such transplantation efforts currently utilize complete microbiome transfers from healthy donors or simple synthetic microbiomes constructed from a small collection of carefully handpicked strains [19], [21], [22]. Yet, to allow the construction of such engineered communities on a large scale and to enable researchers to search the vast space of possible community compositions, a more comprehensive design framework is clearly needed [42]. The development of predictive community models is a critical first step toward such rational design of microbial communities, and will enable researchers, for example, to move from complete microbiome transplantations toward a targeted and personalized microbiome-based therapy [41], [86]. Such a predictive comprehensive framework for modeling microbial communities, however, cannot be gained without a principled understanding of how the joint activity of multiple species influences their environment and vice versa. A recent study, for example, demonstrated how different environmental conditions may induce different forms of species interactions and that it may be the environment, rather than the gain or loss of genes that have larger impact on the specific type of interaction two species will have [48]. Conversely, our study highlights the prevalence of species interactions that will impact the environment via emergent biosynthetic capacities. Our framework not only allows exploring the boundaries of the metabolic tasks microbial consortia can accomplish but also provides mechanistic insights on the pathway level and accounts for the impact of the abundances of species in the community. Moreover, our findings suggest potentially universal principles that have important bearing on community design efforts. For example, the Goldilocks principle observed above points to potentially more promising starting points in the search for communities that exhibit novel biosynthetic capabilities even when relatively little is known about the participating species. Similarly, the contribution of *B. subtilis* to multiple emergent biosynthetic processes demonstrated by our analysis suggests it may be a preferred partner in designed communities, in agreement with its role in promoting plant growth or in maintaining healthy gut communities [69], [87].

Clearly, much work is still ahead before a complete, predictive, and multi-scale framework for modeling microbial communities can be fully realized. Fast and accurate metabolic reconstructions, multi-omics data integration, and the development of novel community-level integration techniques will all contribute tremendously to our ability to model naturally occurring complex ecosystems. Furthermore, in the context of host-associated communities, it will likely be equally important to incorporate a model of the host and its interaction with the community. Yet, our framework and other modeling frameworks of simple microbial communities are an important first step toward the construction of such a comprehensive model. Moreover, these frameworks are already capable of generating testable hypotheses on niche construction and on microbial interaction, further elucidating the forces that govern the assembly, function, and dynamics of microbial ecosystems [25]. Multiple such frameworks could be integrated and ultimately coupled with optimization and design algorithms, resulting in a comprehensive framework for designing novel microbial communities with desired metabolic activities or clinical manipulation of environmental- and host-associated communities.

## Methods

### Flux Balance Analysis

Flux Balance Analysis (FBA) describes cellular fluxes at steady state with the mass balance equation:
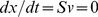
(1)where *x* is the vector of metabolite concentrations, *v* is the vector of reaction fluxes, and *S* is the stoichiometric matrix describing how many molecules are consumed or produced by each reaction. Under this steady state assumption, nutrients acquired from the environment are used to produce biomass or byproducts, with no accumulation of metabolites in the cell. Additional constraints, including reversibility of reactions and measured exchange fluxes, can be incorporated to further limit the possible solution space of metabolic fluxes. Given the complete set of constraints, FBA predicts a specific flux distribution by optimizing a given objective function, typically maximum growth [88]. Growth rate is approximated by a *v^gro^* reaction that consumes energy and a predefined set of nutrients at some relative proportion to form biomass:
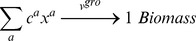
(2)This maximum growth objective has proved successful in providing predictions that are consistent with experimental data [33], [50], [89].

### A Multi-Scale Framework for Community Metabolism Dynamics

Given the set of species that comprise some microbial community and the composition of an initial growth medium, we used a computational framework to characterize the growth of the community on this medium over time. Our framework is a multi-species extension of the dynamic FBA method [50], [51], which aims to predict the temporal behavior of microbial systems (and see also refs [54], [57]). Briefly, we divided the entire growth period into short time intervals (0.1 hr), assuming a steady state solution in each interval and using FBA to obtain the flux distribution and growth rate of each species during this interval. We then calculated the impact of this predicted activity on the community and on the environment at the end of this interval and updated environmental attributes before simulating the next time interval. Specifically, each time step includes these three basic steps:

1. *Determination of uptake rates*: We assume that species grow in a well-mixed environment such that each species could sense nutrient availability, determining the uptake limit for metabolite *j* in the shared environment, *LB^j^*(*t*), with single substrate Michaelis-Menten equations. This formulation guarantees that uptake rates are not sensitive to very low concentrations, preventing sharp shifts in behavior between consecutive time points. Notably, in the FBA models used in this study, the stoichiometric coefficient of nutrient transport reactions is defined as −1 (rather than +1). Accordingly, it is the *lower* bound, rather than the *upper* bound of a transport reaction that determines its uptake limit [31]. Furthermore, we followed the procedure proposed in ref [50] for allocating available metabolites among the various species by estimating an uptake limit for metabolite *j*, *per cell* and *per unit of time*. Specifically, this procedure assumes a scenario wherein metabolite *j* is fully consumed within a single time interval *Δt* by all the cells in the culture. The limit was accordingly set by normalizing the available concentration of metabolite *j* by the total biomass and by the length of the time interval (see second term in equation 3 below). With this limit, only a portion of each metabolite was allocated to each species (proportional to its density in the culture) during each time interval, guaranteeing that a species would not consume nutrients allocated to other species. Furthermore, in the context of our framework, this criterion further assured that the order in which species growth was simulated during each time interval did not impact nutrient availability for other species as nutrients were pre-allocated within each time interval. The smaller of these two fluxes (*i.e.*, the Michaelis-Menten equation and the flux limit per cell per unit of time *Δt*) was used as the uptake limit of nutrient *j*, *LB^j^*, at time point *t*:
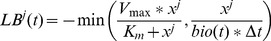
(3)where *x^j^* is the concentration of metabolite *j*, *V_max_* is the maximum velocity to transport metabolite *j*, *K_m_* is the affinity of the transporter, and *bio*(*t*) is the total biomass of all species. Since genome-scale Michaelis-Menten parameters are not yet available, we followed a previous study [90] in using a universal uptake limit and setting the maximum velocity, *V_max_* = 20 for all reactions (except H_2_O and phosphate for which *V_max_* = 1000; see below). For transporter affinity, we used a universal intermediate value (based on available values in the Brenda database; [91], [92]), setting *K_m_* to 0.05. We further followed typical dynamic FBA studies in simulating a batch culture growth rather than a chemostat setting to avoid estimating chemostat parameters. All initial concentrations were set to 10 mM, except for H_2_O and phosphate which most FBA studies assume are unlimited and were therefore set to 10^6^.
2. *Determination of metabolic fluxes via FBA*: Once the maximum nutrient uptake rates *LB*(*t*) were calculated in step 1, we determined the flux distribution and growth rate of each species in the current time interval *Δt*. As discussed in the introduction, we assume that each species *k* aims to maximize its own growth, using FBA to determine its metabolic activity at time point *t* with the following constraints:
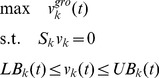
(4)The lower bound of each exchange reaction *j*, *LB^j^*(*t*) was determined as described above according to nutrient availability in the medium at time *t*. For compatibility with previous studies that used these single species models, the lower bound of other intracellular, non-exchange fluxes and the upper bound *UB^j^*(*t*) of all reactions were defined as in the original single-species models. Specifically, in such models, unconstrained fluxes are often marked as having some default arbitrarily large number as bound. To maintain compatibility, we therefore used the same default limits as those defined in the studies from which the models were obtained (and these default limits are also included in the models we provide on our website as indicated below). We confirmed that using a uniform limit across all models yields basically identical results (variation in emergence was <0.003%). To obtain a single and consistent flux solution among all possible alternative solutions that yield the same optimal growth, we further minimized the total flux activity (*i.e.*, the sum of absolute fluxes subject to the predicted optimal growth rate). This procedure assumes that organisms prefer to operate their metabolism with minimal enzymatic cost as proposed in ref. [93], and is a common second optimization step for selecting a unique flux solution. FBA optimization was performed with the GNU Linear Programming Kit (GLPK; http://www.gnu.org/software/glpk/) using the GLPKmex Matlab interface (http://glpkmex.sourceforge.net/).
3. *Environment and community updating*: Given the predicted flux activity and growth rates, we assume that each species *k* grows exponentially with a constant rate *μ_k_* = *v_k_^gro^* (*t_1_*) over the time interval *Δt* = *t_2_*−*t_1_*. The biomass of species *k* at the next time point *t_2_* was then calculated by:
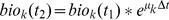
(5)

The concentration of metabolite *j* in the medium, *x^j^*, was updated according to the biomass *bio_k_*(*t_1_*), the growth rate *μ_k_*, and the exchange flux *v_k_^j^* of all species *k* in the community over *Δt*, using an equation derived by integrating the differential equation *dx^j^*/*dt* = ∑ *v_k_^j^* *bio_k_(*t_1_*) over time (see detailed derivation of this equation in Text S1):
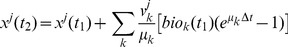
(6)Steps 1–3 were repeated until all of the species in the ecosystem stopped growing in this batch condition. The dynamics of growth, exchange fluxes of all species, as well as the concentrations of all the metabolites in the medium were recorded.

A similar protocol was used to simulate the growth of the two single-species mono-culture systems. The initial biomass concentration in the two mono-cultures was set to be the same as that of the co-culture (0.01 g/Liter) such that the initial nutrient uptake rates are comparable.

### Simple Protocol for Detecting Emergent Metabolites

Once the dynamics of the co-cultures and mono-cultures were simulated, the obtained concentrations of the various metabolites over time were used to detect emergent metabolites. Given a specific growth period (e.g., from beginning of growth to the 1 hr time point), an emergent metabolite was defined as a secreted metabolite whose concentration in the medium exceeds a detection threshold (0.001 mM) at some point during this period in co-culture, but not in either of the two mono-cultures. Note that this definition also naturally excluded metabolites that were present in the initial growth medium.

### Stringent Protocol for Detecting Emergent Metabolites

As described in the main text, FBA provides only a single solution, whereas multiple alternative solutions may exist. Assuming a single solution in each time point while simulating the growth of a given mono-culture can clearly underestimate the scope of metabolites that this mono-culture may secrete to the medium, and consequently lead to spurious prediction of emergent metabolites. To account for all alternative solutions in each time point and for their impact on the behavior of the organism in subsequent time points, we developed an *Iterative Flux Variability Analysis*, extending the previously introduced *Flux Variability Analysis* (FVA) [94]. Briefly, FVA infers the full set of metabolites that *can* be secreted while still satisfying the maximum growth criterion. This is done by attempting to maximize the secretion rate of each metabolite subject to the optimal growth rate calculated via FBA and examining whether the obtained maximized flux is positive. As an iterative extension of this method, we performed FVA in each time point, and took into account possible secretions identified by FVA when updating the concentration of metabolites in the growth medium before simulating the next time interval. To avoid simulating the exponentially growing set of possibilities, we assume that all possible metabolic secretions were in fact secreted *simultaneously* to the medium at the maximal rate without compromising growth. This protocol therefore provided an upper bound for the scope of metabolites that may be secreted by the mono-culture throughout the growth period.

Using this large set of metabolites potentially secreted by the mono-cultures to exclude candidate emergent metabolites accordingly provided a stringent criterion for emergent biosynthetic capacity, potentially underestimating the set of emergent metabolites. In contrast, results obtained with the simple FBA-based protocol described above could overestimate the prevalence of emergent capacity as some identified emergent metabolites are solution-dependent and may not be inferred when alternative solutions are considered. Figure S7 illustrates the differences and similarities between the set of predicted emergent metabolites obtained by these two protocols, highlighting emergent events that were identified by the simple FBA-based protocols and that were removed when the more stringent FVA-based protocol was used. Interestingly, many events of emergent succinate secretions were filtered out by the FVA-based protocol, suggesting that succinate secretion was often found as an alternative optimal solution in mono-culture with potentially higher enzymatic cost. Overall, however, the prevalence level of emergent metabolites was consistent across the two protocols. Throughout the text we aimed to focus on high-confidence predictions of emergent capacity and reported results obtained by the stringent FVA-based protocol.

### Toy Ecosystem Model

The toy ecosystem model described in the main text (and see Figure 2) was defined as follows:

***Red Species:***

Mass balance constraints

*dA/dt = −R_1_−J_1_ = 0*

*dD/dt = R_1_−3R_2_−R_4_ = 0*

*dZ/dt = R_2_−10R_3_+R_4_ = 0*

*dW/dt = 2R_2_−J_4_ = 0*

*dC/dt = −R_4_−J_2_ = 0*

*dY/dt = R_4_−J_3_ = 0*

Flux constraints

*LB_1_<J_1_<∞*

*LB_2_<J_2_<∞*

*LB_3_<J_3_<∞*

*LB_4_<J_4_<∞*

*−∞<R_1_<∞*

*−∞<R_2_<∞*

*−∞<R_3_<∞*

*−∞<R_4_<∞*

***Blue species:***

Mass balance constraints

*dB/dt = −R_5_−J_6_ = 0*

*dX/dt = R_5_−50R_6_ = 0*

*dC/dt = R_5_−J_7_ = 0*

*dA/dt = −R_5_−J_5_ = 0*

Flux constraints

*LB_5_<J_5_<∞*

*LB_6_<J_6_<∞*

*LB_7_<J_7_<∞*

*−∞<R_5_<∞*

*−∞<R_6_<∞*

In the definition above, the *R_i_* denote internal reactions whereas the *J_i_* denote transport reactions. The uptake limits, *LB_i_* for all nutrients were determined as described in eq (3) at each time point. Notably, as indicated by the expressions for d*D*/d*t* and d*Z*/d*t* in the red species, *R_2_* consumes 3 unit of *D* to generate 1 unit of product *Z* whereas *R_4_* requires only 1 unit of *D* to generate 1 unit of *Z*, making *R_4_* a more favorable reaction over *R_2_* as it allows a higher biomass yield per unit of metabolite *D*. Note also that *Y* is secreted as an emergent metabolite once *R_4_* is active (and see also Figure 2).

### Species Models and Neutral Media

We used FBA models from two previously published studies on microbial metabolism. The first set was studied in ref [48] and contained 6 manually curated, high quality species models that span a wide phylogenetic range. This set included models of *Escherichia coli* [95], *Salmonella typhimurium* [96], *Bacillus subtilis* [97], *Methanosarcina barkeri* [98], *Shewanella oneidensis* [99], and an extended genome scale model of *Methylobacterium extorquens* provided by Stephen Van Dien [100]. To ensure compatibility with computationally derived media (see below), we obtained all these models directly from ref [48], using the same versions of the models as in this previous study. We additionally obtained from ref [48] a large scale dataset of computationally derived growth media for each pair of species. These media were classified as either mutualism-inducing media (*i.e.*, media that allow for the growth of both species in co-culture but do not support growth of either species when grown in isolation), commensalism-inducing (*i.e.*, sustain growth of one of the two species but not the other), or neutral (*i.e.*, sustain growth of each species individually). From this dataset, we randomly selected 100 neutral media for each of the two-species communities except for the *Shewanella oneidensis*/*Methanosarcina barkeri* pair for which no neutral media was found in ref [48]. *Helicobacter pylori*, which was also studied in ref [48] had very few neutral media available and was therefore not included in our analysis. The complexity of these media may depend on the models included in each community, but overall, these minimal neutral media contained on average 25.5±5.9 metabolites. Roughly 12∼15 of these compounds were inorganic ions (e.g. Zn^2+^, cobalt^2+^) that are essential and defined as biomass components.

The second set included 116 automatically reconstructed models from the SEED pipeline [37] and was obtained from ref [44] (Table S1). Since a large set of neutral media for communities composed of these SEED models was not available, we applied a mixed-integer linear programming (MILP) method, similar to the one described in ref [44], to infer minimal neutral media for each community. This was done by finding a minimal set of metabolites that supports the growth (above some minimal rate) of each species in isolation, out of a large initial set of nutrients composed of the union of all transportable metabolites of the two species. Conceptually, the objective of this MILP problem was to remove as many nutrients as possible from the initial set (and hence leaving as few as possible in the medium), while maintaining the primary constraint of allowing the growth rate of each species to be larger than some minimal threshold (set here to be *μ_k_*≥0.3). To formulate this objective, we first defined a binary decision variable *θ* for each nutrient, denoting whether this nutrient was removed from the medium or not (*i.e.*, when *θ* = 1 the nutrient was removed from the medium and when *θ* = 0 the nutrient was included). Next, we added a constraint for the uptake flux of nutrient *i*, *v_k_^i^*, connecting the MILP objective with the minimal growth constraint of each species *k*. The aim of this constraint was to guarantee that when *i* is removed from the medium (*i.e.*, *θ_k_^i^* = 1), the nutrient uptake flux *v_k_^i^* was non negative (note that a non-negative *v_k_^i^* constraint means this transporter could not serve as an uptake flux) and that when nutrient *i* was included in the medium (*θ_k_^i^* = 0), *v_k_^i^* could take any value and species *k* was able to take up and utilize metabolite *i*. Mathematically, this concept was implemented with the constraint *v_k_^i^+Lθ_k_^i^*≥*L*, where *L* is a large negative number (here set to *−1000*). This constraint then became *v_k_^i^*≥0 when *θ_k_^i^* = 1, indicating that this transport flux cannot be used for uptake. When *θ_k_^i^* = 0, this constraint became *v_k_^i^*≥*L*, allowing this flux to serve as an uptake flux (e.g. *v_k_^i^* = −10≥*L*). The MILP problem was solved with a single optimization procedure considering the minimal growth constraints of the two species simultaneously. This was done using a stoichiometric matrix that included both species and that described their growth *independently* (*i.e.*, no cross-feeding fluxes were included in the model). Formally, the MILP problem could then be formulated as a maximization of the sum of decision variables as follows:
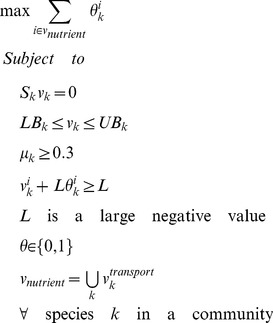
(7)Given the solution of this MILP problem, the set of nutrients for which the decision variable *θ* equals zero in at least one species represented the minimal neutral media. The solution to this MILP problem was determined using the GLPK solver through GLPKmex (see above).

Notably, simulating the growth of all pair-wise communities from the first dataset, each on 100 different media, took ∼2 days on a 144 node cluster. The second dataset included roughly 500 times more pair-wise communities and therefore each community was simulated on one medium as described above. For consistency, in analyzing this second dataset, we only considered communities in which both species were still growing at 1 hr.

All models and media used in this study are available for download on our website (http://elbo.gs.washington.edu/download.html).

### Computing Functional and Phylogenetic Distances

In order to determine the functional and phylogenetic distance among pairs of species, KEGG orthology (KO) annotations [101] and 16S rRNA gene sequence information were obtained from the Integrated Microbial Genome Database (IMG) [102]. Functional distance was calculated as the pairwise Jaccard distance between the KO presence/absence profiles of the two species. Phylogenetic distance was calculated as described in [25] and [103]. Briefly, 16S rRNA sequences were first aligned using NAST [104]. When multiple sequences in a single species passed the NAST filter, a single sequence was chosen at random. Lane mask was applied to this alignment, and percent identity was calculated with Clearcut [105] using the Kimura two-parameter distance correction.

## Supporting Information

Figure S1 Producers of the most prevalent emergent metabolites and their partners. The size of each pie chart reflects the frequency of the corresponding emergent metabolite among the 1400 community/medium simulations.
(TIF)

Figure S2 Metabolic reprogramming in *E. coli* that resulted in emergent ethanol secretion to maintain redox balance. The cross-feeding metabolite fumarate enhanced energy production in the TCA cycle and induced a series of flux reroutes, including ethanol production. Abbreviations – etoh: ethanol; acald: acetaldehyde; ac: acetate; accoa: acetyl-CoA; cit: citrate; icit: Isocitrate; akg: 2-oxoglutarate; succoa: succinyl-CoA; succ: succinate; fum: fumarate; mal-L: L-malate; oaa: oxaloacetate; NAD: nicotinamide adenine dinucleotide; pep: phosphoenolpyruvate; pyr: pyruvate; damp: dAMP.
(TIF)

Figure S3 Metabolic reprogramming in an *E. coli*/*B. subtilis* community, resulting in emergent secretion of both succinate and urea. Abbreviations - etoh: ethanol; acald: acetaldehyde; ac: acetate; accoa: acetyl-CoA; cit: citrate; icit: Isocitrate; akg: 2-oxoglutarate; succoa: succinyl-CoA; succ: succinate; fum: fumarate; mal-L: L-malate; oaa: oxaloacetate; NAD: nicotinamide adenine dinucleotide; pep: phosphoenolpyruvate; pyr: pyruvate; damp: dAMP; lac: L-lactate: glu: glutamate; orn: ornithine; citr: citrulline; arguc: arginosuccinate; arg: arginine.
(TIF)

Figure S4 Emergent metabolites detected when the entire growth period was considered. As in Figure 4, rows represent emergent metabolites (ranked by prevalence) and columns represent specific community/medium combinations. Emergent secretion events detected at 1 hr (*i.e.* those that are included also in Figure 4) are illustrated as black bars. Emergent secretion events (and emergent metabolites) that were detected only when the entire growth period was considered but not in the early growth period are labeled in blue.
(TIF)

Figure S5 Overall consistency in emergent biosynthetic capacity detected in early and late growth. (**A**) The average number of emergent metabolites for each pair-wise species community across 100 media detected at the 1 hr time point (x axis) vs. the average number of emergent metabolites detected when the entire growth period was considered (y axis). A linear regression line is illustrated in red. (**B**) The frequency at which each emergent metabolite was detected at 1 hr vs. the entire growth period (with a linear regression line illustrated). Only metabolites that were detected also at the 1 hr time point are included in this plot.
(TIF)

Figure S6 The Goldilocks principle of emergent biosynthetic capacity observed in simple two-species communities. The plot details are as described in Figure 7, using phylogenetic distance rather than functional distance to measure the distance between community members. Phylogenetic distance was calculated according to the divergence in the 16S rRNA gene (Methods).
(TIF)

Figure S7 Emergent metabolites predicted by the simple FBA-based protocol (and compare with Figure 4). Predictions were made at the early growth period (<1 hr). The black bars represent predictions that were verified also by the stringent FVA-based protocol (and that are therefore included in Figure 4). The magenta bars are additional predictions made by the FBA-based protocol and that were filtered by the more stringent FVA-based protocol. The prevalence of each emergent metabolite predicted by the FVA- and FBA-based protocols is shown in parenthesis (e.g. ethanol was predicted in only 158 community/media configurations by the FVA-based protocol but in 170 configurations by the FBA-based protocol). Note that, by definition, FVA-based predictions are a subset of FBA-based predictions. Similarly, the number of media in which at least one emergent metabolite was detected for each species composition and with each of the two protocols is listed on the top.
(TIF)

Figure S8 Growth advantage in co-culture compared to mono-culture for (**A**) all species in all community/medium settings; (**B**) producers in community/medium settings that exhibited emergent biosynthetic capacity; and (**C**) partners in community/medium settings that exhibited emergent biosynthetic capacity. (**D**) Community-level growth advantage, comparing the total biomass of the co-culture to the combined biomass of the two mono-cultures.
(TIF)

Figure S9 The growth penalty associated with secretion of emergent metabolites. The distribution of the relative growth rate of the producer when forced to secrete emergent metabolites in mono-culture compared to its growth rate with no constraints on secretion is illustrated. The leftmost bin represents cases in which the producer is not able to grow when forcing the secretion of the emergent metabolite in mono-culture.
(TIF)

Figure S10 Confirming the Goldilocks principle of emergent biosynthetic capacity in 500 ‘universally neutral’ media (see Text S1). The plot details are as described in Figure 7.
(TIF)

Table S1 A list of all the SEED models used in our study.
(PDF)

Text S1 Supporting information, including a derivation of equation (6), an analysis of the impact of emergent biosynthetic capacity on growth, an analysis of the role of the partner in emergent biosynthetic capacity, and a validation of the Goldilocks principle in universally neutral media.
(PDF)
