# Supplementary figures and images for: Emergent Biosynthetic Capacity in Simple Microbial Communities
Source: PLoS Comput Biol. 2014 Jul 3;10(7):e1003695. doi: 10.1371/journal.pcbi.1003695 (PMC4084645; doi:10.1371/journal.pcbi.1003695)

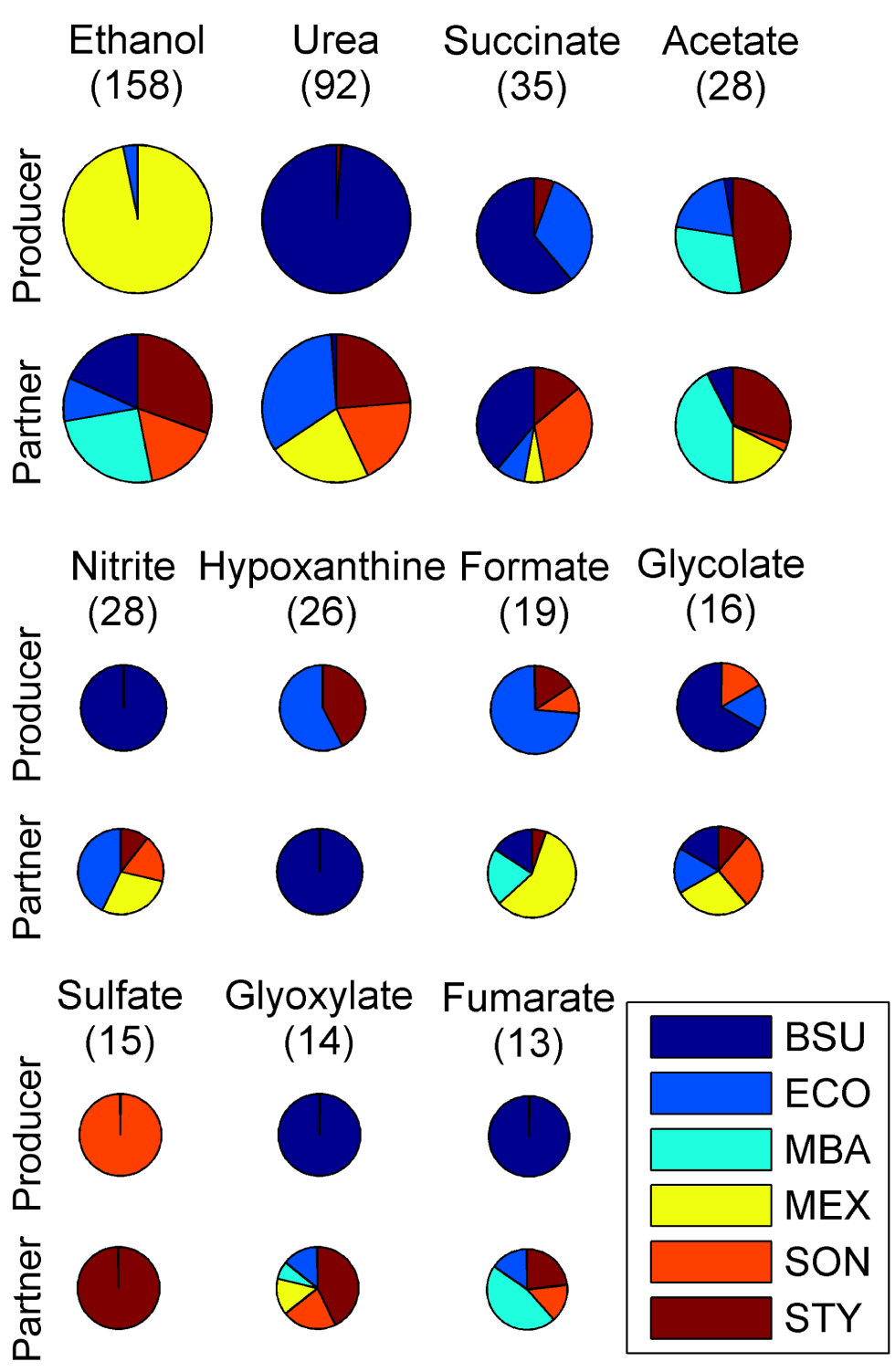

Supplement: Figure S1 — Producers of the most prevalent emergent metabolites and their partners. The size of each pie chart reflects the frequency of the corresponding emergent metabolite among the 1400 community/medium simulations. (TIF) [file pcbi.1003695.s001.tif]

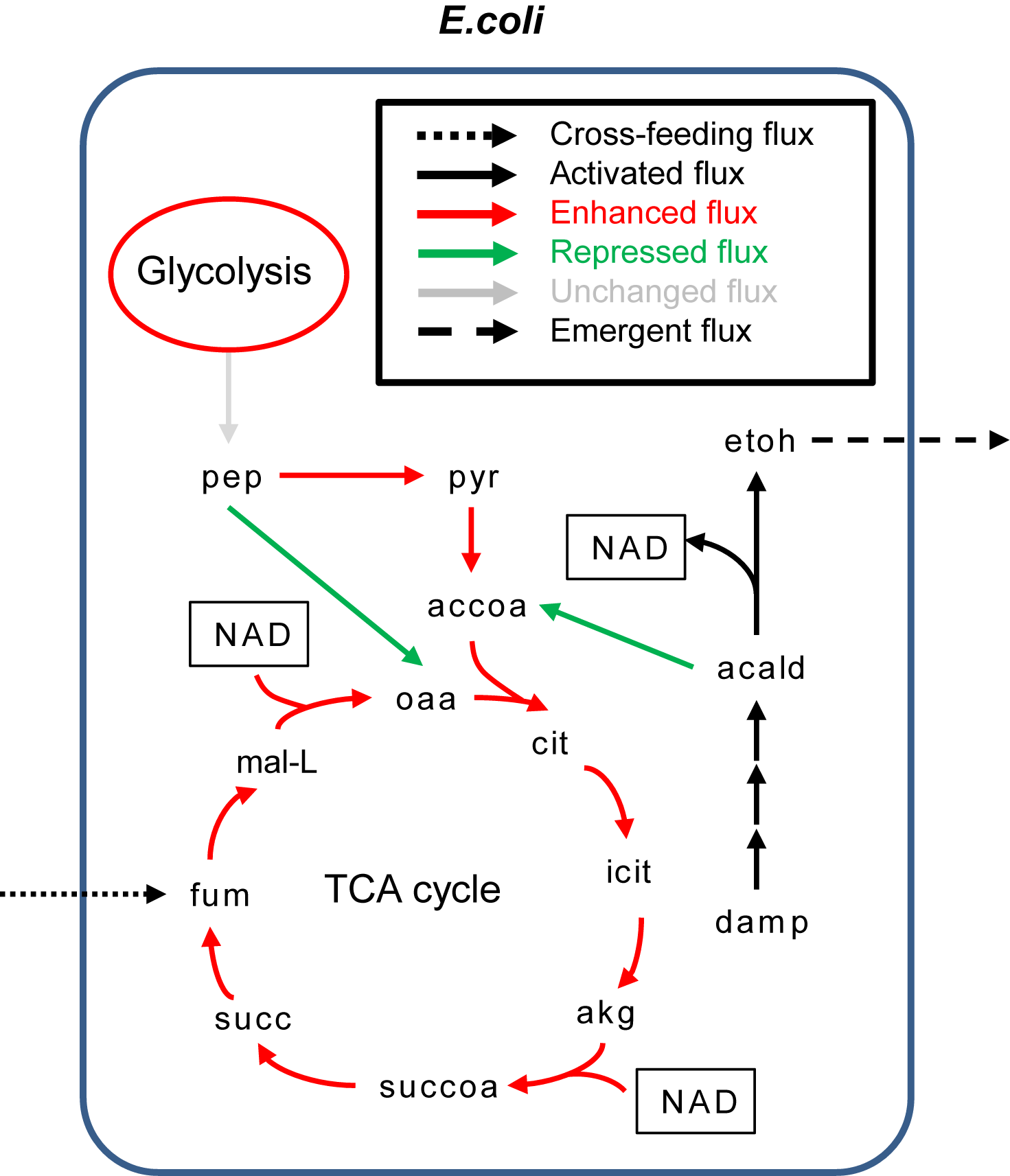

Supplement: Figure S2 — Metabolic reprogramming in E. coli that resulted in emergent ethanol secretion to maintain redox balance. The cross-feeding metabolite fumarate enhanced energy production in the TCA cycle and induced a series of flux reroutes, including ethanol production. Abbreviations – etoh: ethanol; acald: acetaldehyde; ac: acetate; accoa: acetyl-CoA; cit: citrate; icit: Isocitrate; akg: 2-oxoglutarate; succoa: succinyl-CoA; succ: succinate; fum: fumarate; mal-L: L-malate; oaa: oxaloacetate; NAD: nicotinamide adenine dinucleotide; pep: phosphoenolpyruvate; pyr: pyruvate; damp: dAMP. (TIF) [file pcbi.1003695.s002.tif]

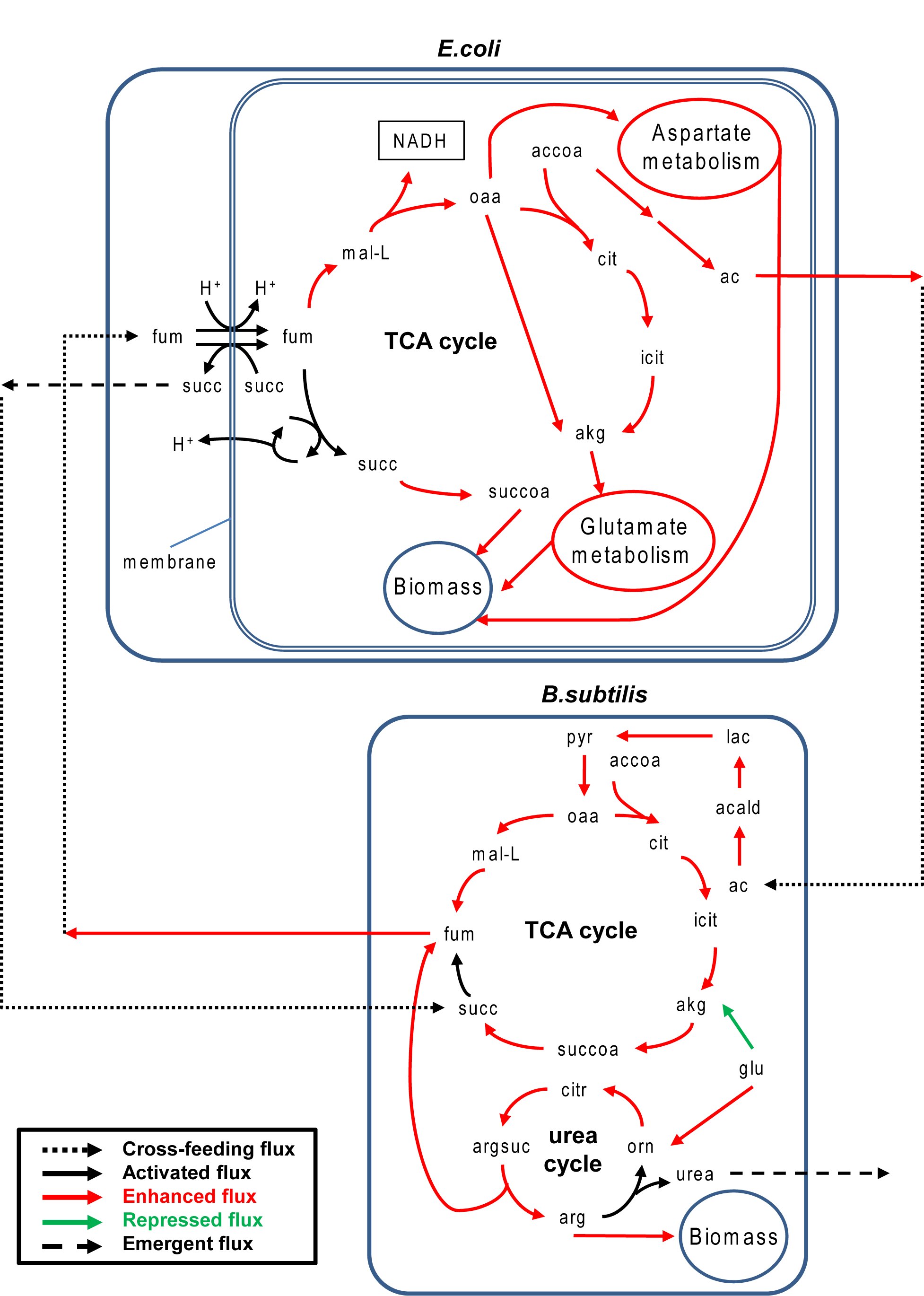

Supplement: Figure S3 — Metabolic reprogramming in an E. coli/B. subtilis community, resulting in emergent secretion of both succinate and urea. Abbreviations - etoh: ethanol; acald: acetaldehyde; ac: acetate; accoa: acetyl-CoA; cit: citrate; icit: Isocitrate; akg: 2-oxoglutarate; succoa: succinyl-CoA; succ: succinate; fum: fumarate; mal-L: L-malate; oaa: oxaloacetate; NAD: nicotinamide adenine dinucleotide; pep: phosphoenolpyruvate; pyr: pyruvate; damp: dAMP; lac: L-lactate: glu: glutamate; orn: ornithine; citr: citrulline; arguc: arginosuccinate; arg: arginine. (TIF) [file pcbi.1003695.s003.tif]

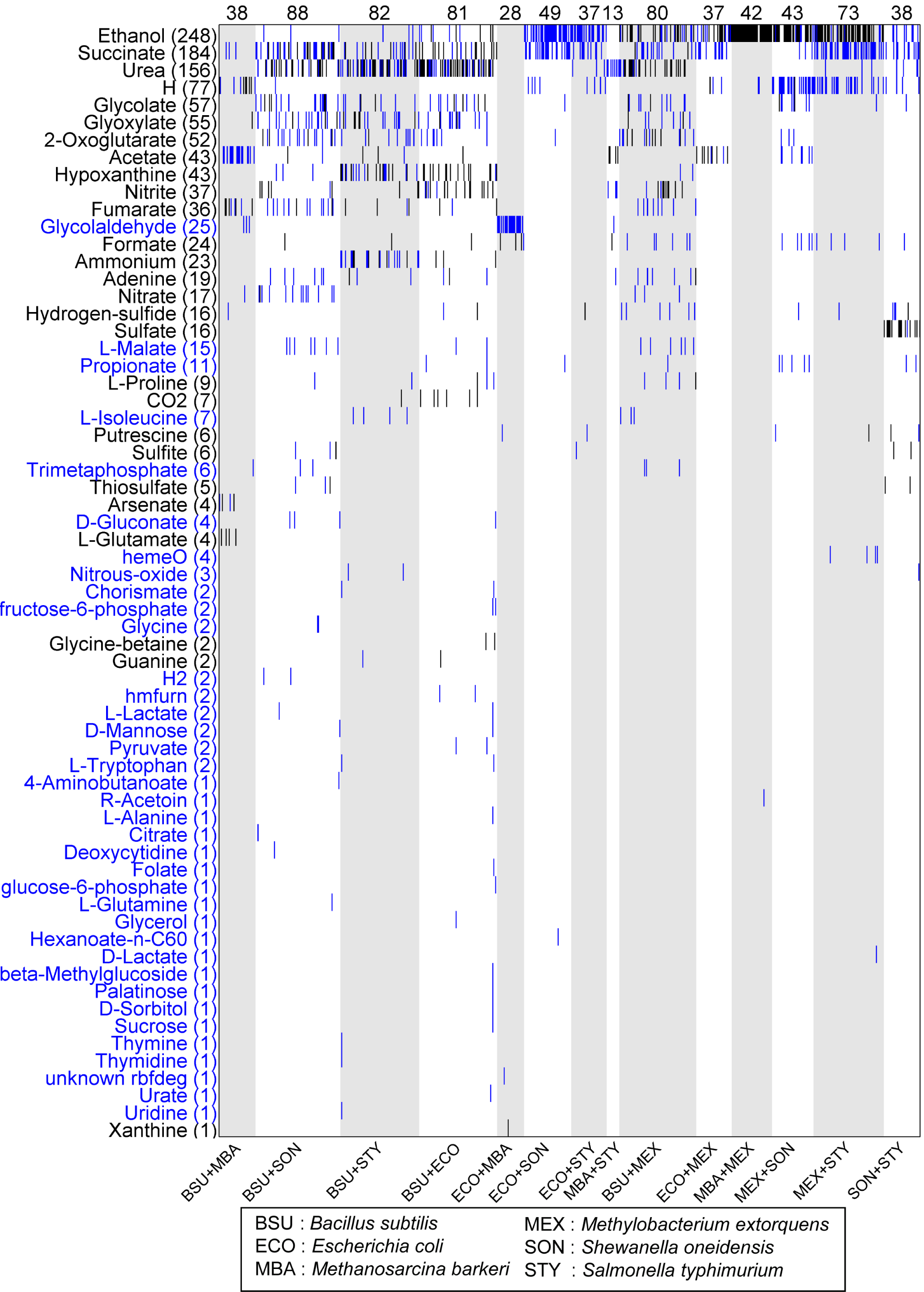

Supplement: Figure S4 — Emergent metabolites detected when the entire growth period was considered. As in Figure 4, rows represent emergent metabolites (ranked by prevalence) and columns represent specific community/medium combinations. Emergent secretion events detected at 1 hr (i.e. those that are included also in Figure 4) are illustrated as black bars. Emergent secretion events (and emergent metabolites) that were detected only when the entire growth period was considered but not in the early growth period are labeled in blue. (TIF) [file pcbi.1003695.s004.tif]

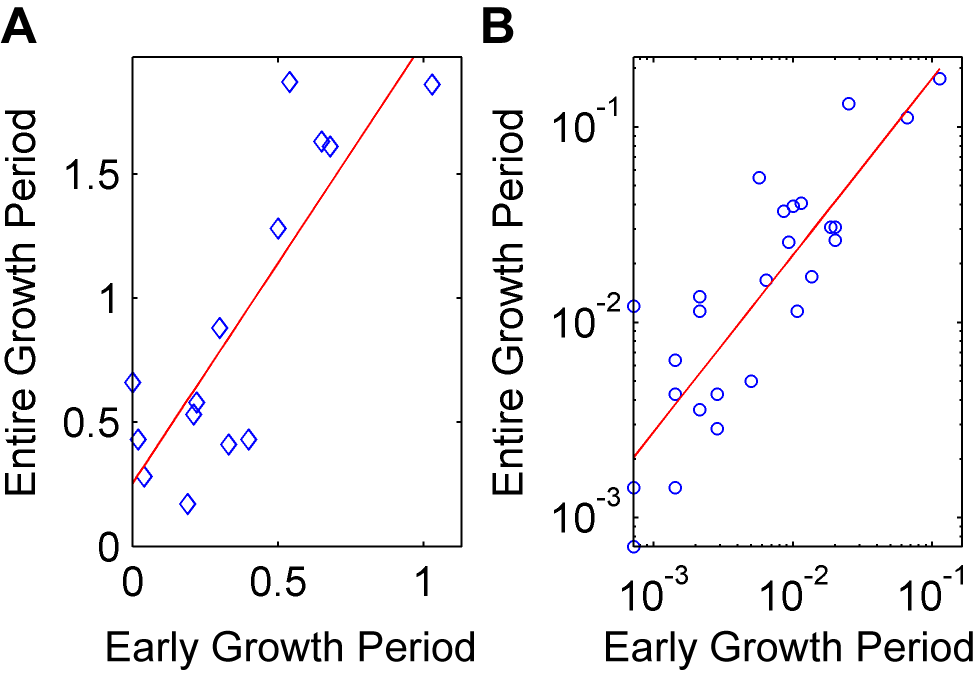

Supplement: Figure S5 — Overall consistency in emergent biosynthetic capacity detected in early and late growth. (A) The average number of emergent metabolites for each pair-wise species community across 100 media detected at the 1 hr time point (x axis) vs. the average number of emergent metabolites detected when the entire growth period was considered (y axis). A linear regression line is illustrated in red. (B) The frequency at which each emergent metabolite was detected at 1 hr vs. the entire growth period (with a linear regression line illustrated). Only metabolites that were detected also at the 1 hr time point are included in this plot. (TIF) [file pcbi.1003695.s005.tif]

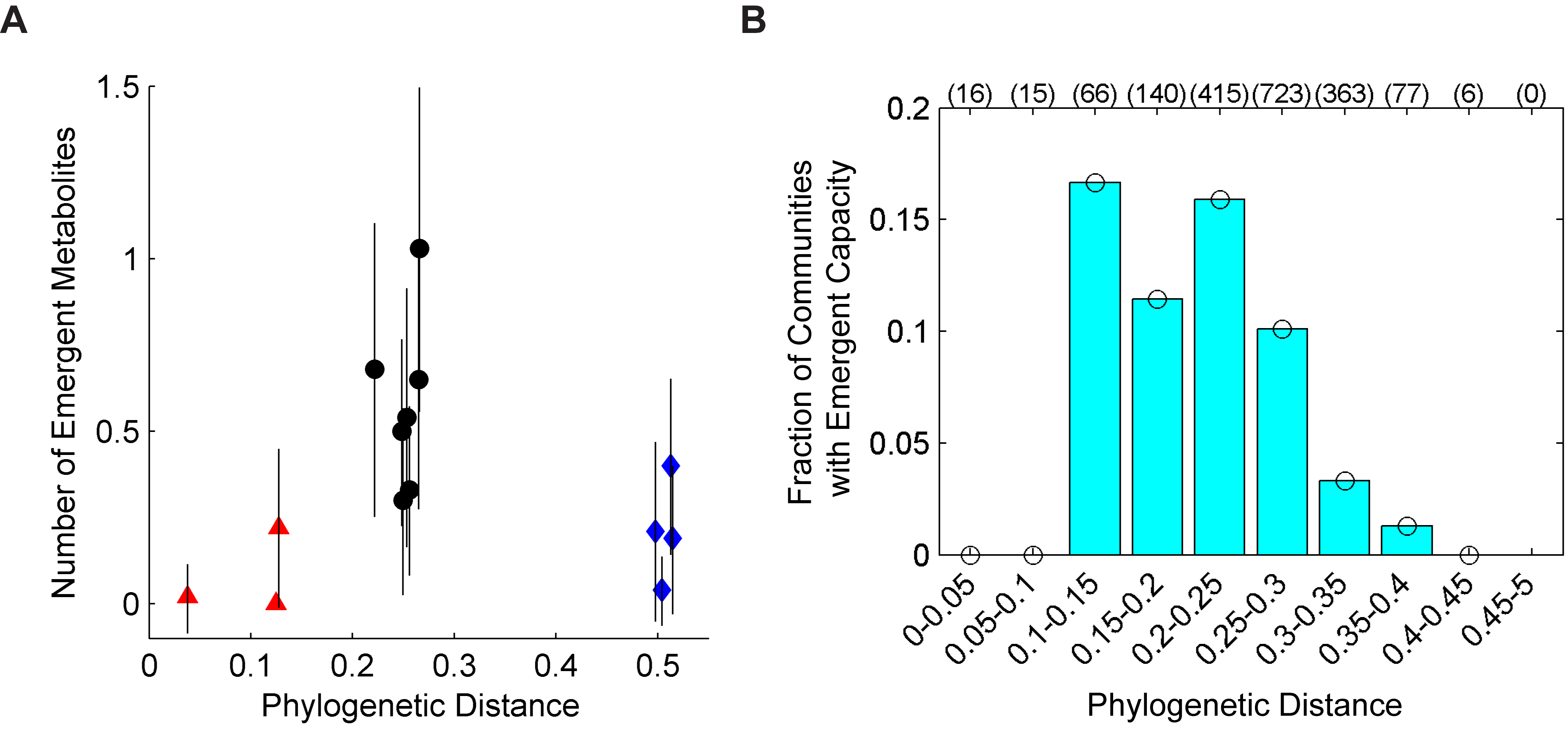

Supplement: Figure S6 — The Goldilocks principle of emergent biosynthetic capacity observed in simple two-species communities. The plot details are as described in Figure 7, using phylogenetic distance rather than functional distance to measure the distance between community members. Phylogenetic distance was calculated according to the divergence in the 16S rRNA gene (Methods). (TIF) [file pcbi.1003695.s006.tif]

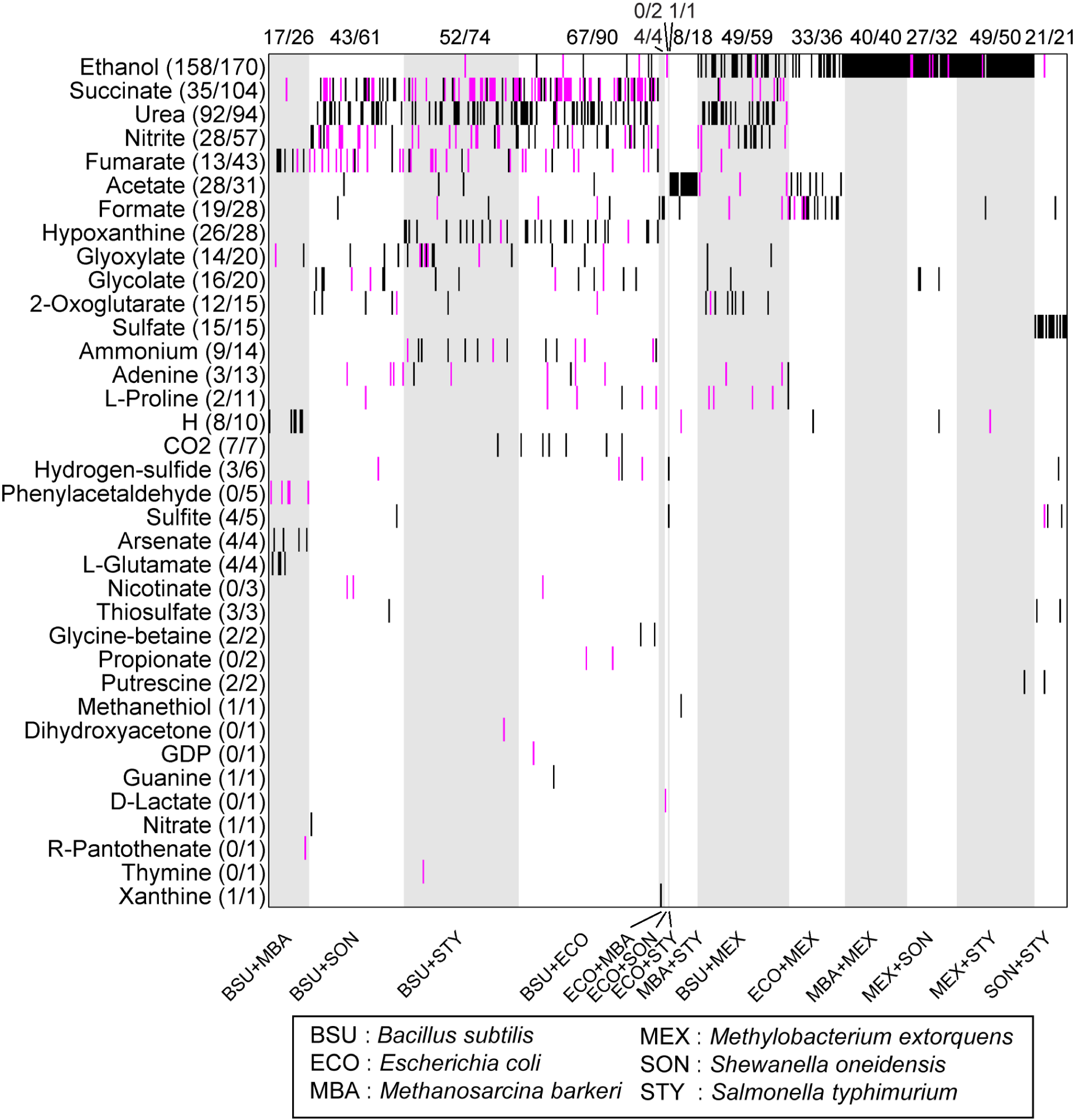

Supplement: Figure S7 — Emergent metabolites predicted by the simple FBA-based protocol (and compare with Figure 4). Predictions were made at the early growth period (<1 hr). The black bars represent predictions that were verified also by the stringent FVA-based protocol (and that are therefore included in Figure 4). The magenta bars are additional predictions made by the FBA-based protocol and that were filtered by the more stringent FVA-based protocol. The prevalence of each emergent metabolite predicted by the FVA- and FBA-based protocols is shown in parenthesis (e.g. ethanol was predicted in only 158 community/media configurations by the FVA-based protocol but in 170 configurations by the FBA-based protocol). Note that, by definition, FVA-based predictions are a subset of FBA-based predictions. Similarly, the number of media in which at least one emergent metabolite was detected for each species composition and with each of the two protocols is listed on the top. (TIF) [file pcbi.1003695.s007.tif]

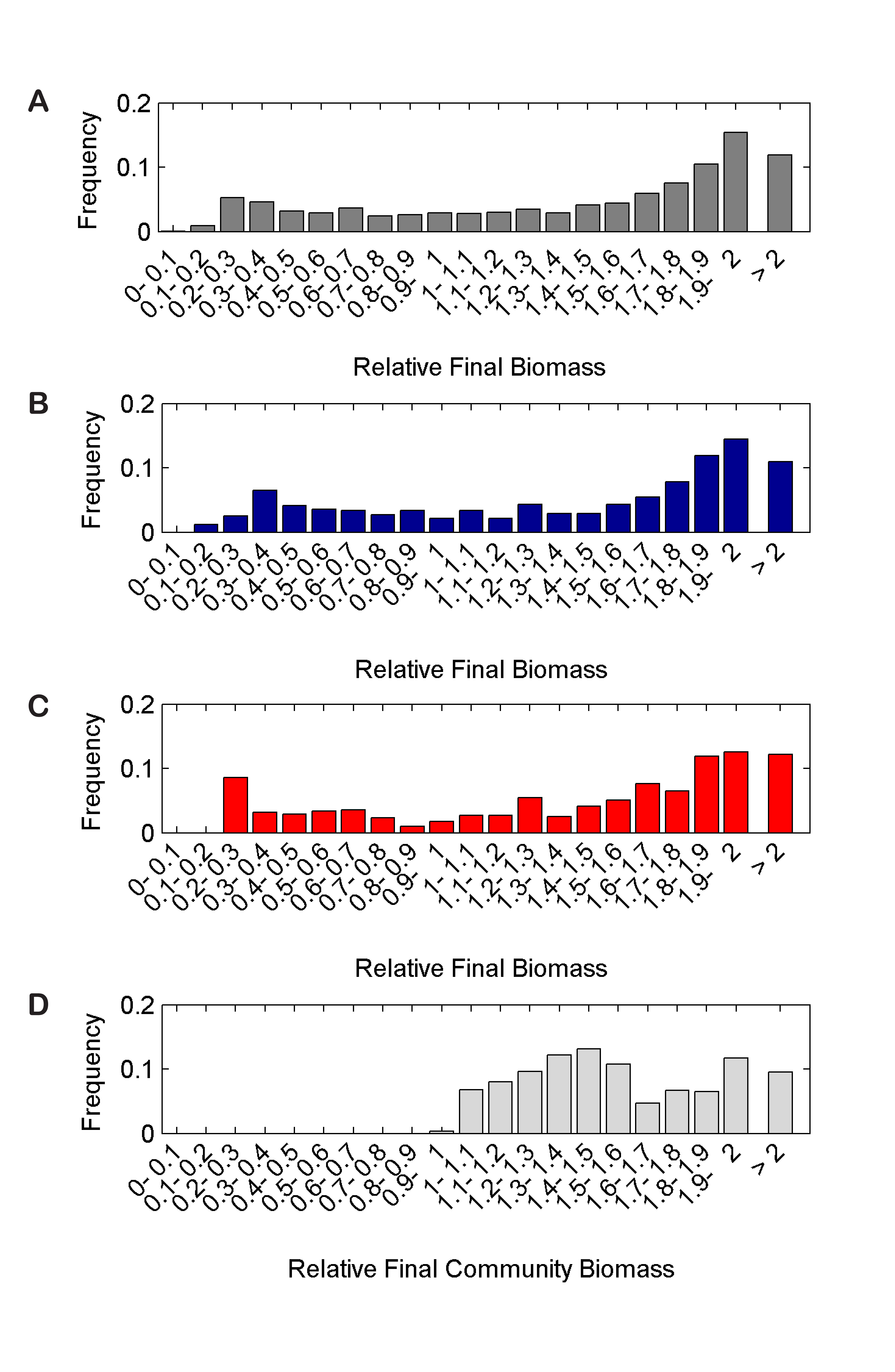

Supplement: Figure S8 — Growth advantage in co-culture compared to mono-culture for (A) all species in all community/medium settings; (B) producers in community/medium settings that exhibited emergent biosynthetic capacity; and (C) partners in community/medium settings that exhibited emergent biosynthetic capacity. (D) Community-level growth advantage, comparing the total biomass of the co-culture to the combined biomass of the two mono-cultures. (TIF) [file pcbi.1003695.s008.tif]

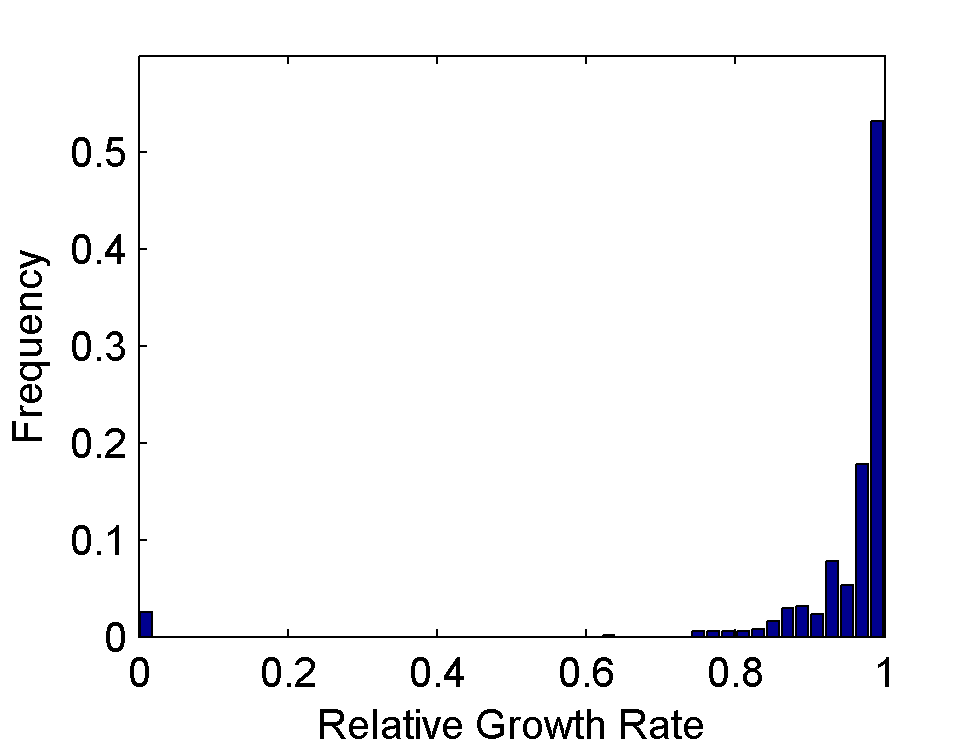

Supplement: Figure S9 — The growth penalty associated with secretion of emergent metabolites. The distribution of the relative growth rate of the producer when forced to secrete emergent metabolites in mono-culture compared to its growth rate with no constraints on secretion is illustrated. The leftmost bin represents cases in which the producer is not able to grow when forcing the secretion of the emergent metabolite in mono-culture. (TIF) [file pcbi.1003695.s009.tif]

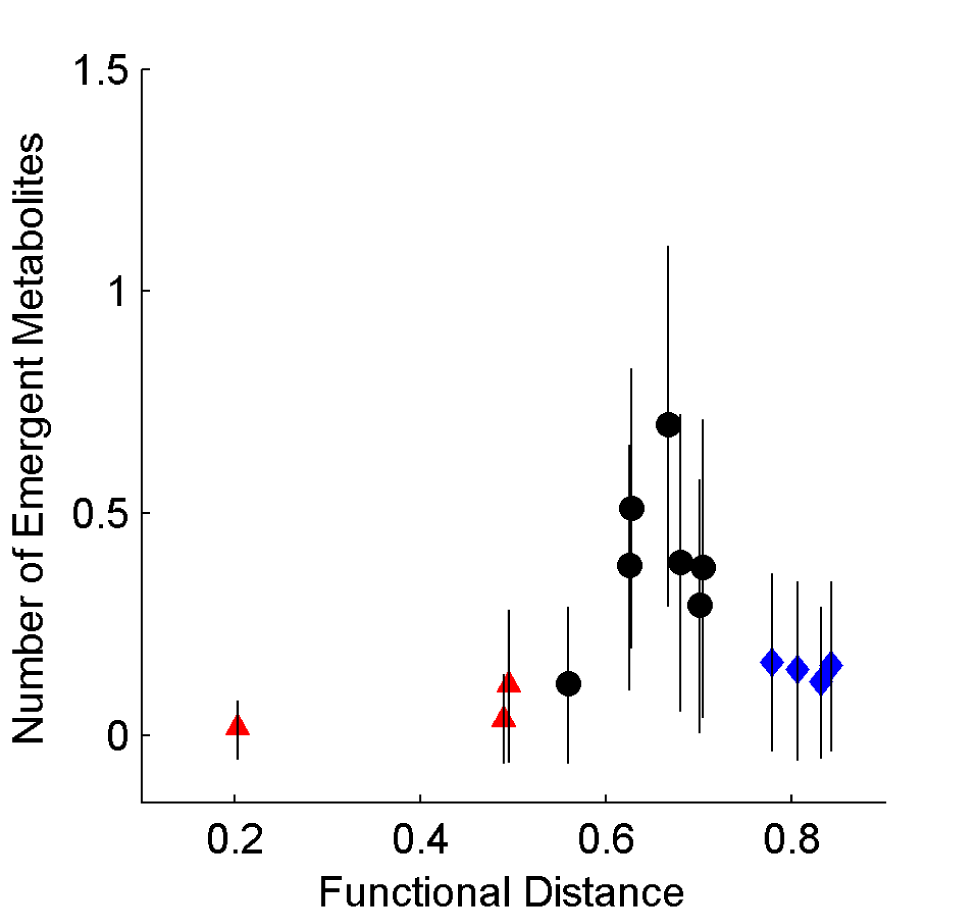

Supplement: Figure S10 — Confirming the Goldilocks principle of emergent biosynthetic capacity in 500 ‘universally neutral’ media (see Text S1). The plot details are as described in Figure 7. (TIF) [file pcbi.1003695.s010.tif]
