# Supplementary material for: Emergent Biosynthetic Capacity in Simple Microbial Communities
Source: PLoS Comput Biol. 2014 Jul 3;10(7):e1003695. doi: 10.1371/journal.pcbi.1003695 (PMC4084645; doi:10.1371/journal.pcbi.1003695)
Supplement: Table S1 — A list of all the SEED models used in our study. (PDF) [file pcbi.1003695.s011.pdf]

Table S1: SEED models used in the study

SEED Model Name	SEED ID	IMG Genome Name / Sample Name
Acinetobacter sp. ADP1	Core62977_3	Acinetobacter baylyi ADP1
Anaeromyxobacter dehalogenans 2CP- C	Core290397_13	Anaeromyxobacter dehalogenans 2CP-C
Anaplasma marginale str. St. Maries	Core234826_3	Anaplasma marginale St. Maries
Aquifex aeolicus VF5	Core224324_1	Aquifex aeolicus VF5
Bacillus anthracis str. Ames Ancestor	Core261594_1	Bacillus anthracis Ames Ancestor A2084
Bacteroides fragilis YCH46	Core295405_3	Bacteroides fragilis YCH46
Bdellovibrio bacteriovorus HD100	Core264462_1	Bdellovibrio bacteriovorus HD100
Bifidobacterium longum NCC2705	Core206672_1	Bifidobacterium longum NCC2705
Bordetella pertussis Tohama I	Core257313_1	Bordetella pertussis Tohama I
Borrelia burgdorferi B31	Core224326_1	Borrelia burgdorferi B31
Bradyrhizobium japonicum USDA 110	Core224911_1	Bradyrhizobium japonicum USDA 110
Brucella melitensis 16M	Core224914_1	Brucella melitensis bv 1, 16M
Buchnera aphidicola str. APS (Acyrthosiphon pisum)	Core107806_1	Buchnera aphidicola APS
Burkholderia pseudomallei K96243	Core272560_3	Burkholderia pseudomallei K96243
Campylobacter jejuni subsp. jejuni NCTC 11168	Core192222_1	Campylobacter jejuni jejuni NCTC 11168
Blochmannia floridanus	Core203907_1	Candidatus Blochmannia floridanus
Carboxydothermus hydrogenoformans Z-2901	Core246194_3	Carboxydothermus hydrogenoformans Z-2901
Caulobacter crescentus CB15	Core190650_1	Caulobacter crescentus CB15
Chlamydia trachomatis D/UW-3/CX	Core272561_1	Chlamydia trachomatis D/UW-3/CX (sv D)
Chlamydophila pneumoniae AR39	Core115711_7	Chlamydophila pneumoniae AR39
Clostridium acetobutylicum ATCC 824	Core272562_1	Clostridium acetobutylicum ATCC 824
Clostridium tetani E88	Core212717_1	Clostridium tetani Massachusetts E88
Coxiella burnetii RSA 493	Core227377_1	Coxiella burnetii Nine Mile phase I / RSA 493
Cytophaga hutchinsonii ATCC 33406	Core269798_12	Cytophaga hutchinsonii ATCC 33406
Dehalococcoides ethenogenes 195	Core243164_3	Dehalococcoides ethenogenes 195
Desulfovibrio desulfuricans G20	Core207559_3	Desulfovibrio alaskensis G20
Ehrlichia ruminantium str. Gardel	Core302409_3	Ehrlichia ruminantium Gardel
Escherichia coli W3110	Core316407_3	Escherichia coli K12- W3110
Francisella tularensis subsp. tularensis Schu 4	Core177416_3	Francisella tularensis tularensis CHU S4
Frankia sp. Ccl3	Core106370_11	Frankia sp. CcI3
Gluconobacter oxydans 621H	Core290633_1	Gluconobacter oxydans 621H
Haemophilus influenzae Rd KW20	Core71421_1	Haemophilus influenzae Rd (KW20)
Helicobacter pylori 26695	Core85962_1	Helicobacter pylori 26695
Idiomarina loihiensis L2TR	Core283942_3	Idiomarina loihiensis L2TR
Lactobacillus plantarum WCFS1	Core220668_1	Lactobacillus plantarum WCFS1
Lactococcus lactis subsp. lactis Il1403	Core272623_1	Lactococcus lactis lactis Il1403

Legionella pneumophila subsp. pneumophila str. Philadelphia 1	Core272624_3	Legionella pneumophila pneumophila Philadelphia-1
Leifsonia xyli subsp. xyli str. CTCB07	Core281090_3	Leifsonia xyli xyli CTCB07
Leptospira interrogans serovar Copenhageni str. Fiocruz L1-130	Core267671_1	Leptospira interrogans sv Copenhageni Fiocruz L1-130
Listeria innocua Clip11262	Core272626_1	Listeria innocua Clip11262
Listeria monocytogenes EGD-e	Core169963_1	Listeria monocytogenes EGD-e
Magnetospirillum magneticum AMB-1	Core342108_5	Magnetospirillum magneticum AMB-1
Mannheimia succiniciproducens MBEL55E	Core221988_1	Basfia succiniciproducens MBEL55E
Methylobacillus flagellatus KT	Core265072_7	Methylobacillus flagellatus KT
Methylococcus capsulatus str. Bath	Core243233_4	Methylococcus capsulatus Bath
Mycobacterium tuberculosis H37Rv	Core83332_1	Mycobacterium tuberculosis H37Rv (lab strain)
Mycoplasma genitalium G-37	Core243273_1	Mycoplasma genitalium G37
Mycoplasma pulmonis UAB CTIP	Core272635_1	Mycoplasma pulmonis UAB CTIP
Neisseria gonorrhoeae FA 1090	Core242231_4	Neisseria gonorrhoeae FA 1090
Neisseria meningitidis MC58	Core122586_1	Neisseria meningitidis MC58
Nitrosococcus oceani ATCC 19707	Core323261_3	Nitrosococcus oceani C-107, ATCC 19707
Nitrosomonas europaea ATCC 19718	Core228410_1	Nitrosomonas europaea ATCC 19718
Nocardia farcinica IFM 10152	Core247156_1	Nocardia farcinica IFM 10152
Onion yellows phytoplasma OY-M	Core262768_1	Candidatus Phytoplasma onion yellows OY-M
Photobacterium profundum SS9	Core298386_1	Photobacterium profundum SS9
Photorhabdus luminescens subsp. laumondii TTO1	Core243265_1	Photorhabdus luminescens laumondii TTO1
Polaromonas sp. JS666	Core296591_1	Polaromonas sp. JS666
Pseudoalteromonas haloplanktis TAC125	Core326442_4	Pseudoalteromonas haloplanktis TAC125
Pseudomonas aeruginosa PAO1	Core208964_1	Pseudomonas aeruginosa PAO1
Pseudomonas fluorescens PfO-1	Core205922_3	Pseudomonas fluorescens Pf0-1
Pseudomonas putida KT2440	Core160488_1	Pseudomonas putida KT2440
Ralstonia solanacearum GMI1000	Core267608_1	Ralstonia solanacearum GMI1000
Rhodopseudomonas palustris CGA009	Core258594_1	Rhodopseudomonas palustris CGA009
Rickettsia prowazekii str. Madrid E	Core272947_1	Rickettsia prowazekii Madrid E
Rubrobacter xylanophilus DSM 9941	Core266117_6	Rubrobacter xylanophilus DSM 9941
Salinibacter ruber DSM 13855	Core309807_5	Salinibacter ruber M31, DSM 13855
Salmonella typhimurium LT2	Core99287_1	Salmonella enterica enterica sv Typhimurium LT2 LT2
Shewanella frigidimarina NCIMB 400	Seed318167_10	Shewanella frigidimarina NCIMB 400
Shewanella oneidensis MR-1	Seed211586_1	Shewanella oneidensis MR-1
Shigella flexneri 2a str. 2457T	Core198215_1	Shigella flexneri 2a 2457T
Sinorhizobium meliloti 1021	Core266834_1	Ensifer meliloti 1021
Staphylococcus aureus subsp. aureus COL	Core93062_4	Staphylococcus aureus aureus COL
Staphylococcus aureus subsp. aureus Mu50	Core158878_1	Staphylococcus aureus aureus M
Staphylococcus aureus subsp. aureus N315	Core158879_1	Staphylococcus aureus aureus N315

Staphylococcus aureus subsp. aureus NCTC 8325	Core93061_3	Staphylococcus aureus aureus NCTC 8325
Streptococcus pneumoniae R6	Core171101_1	Streptococcus pneumoniae R6
Streptococcus pneumoniae TIGR4	Core170187_1	Streptococcus pneumoniae TIGR4
Streptococcus thermophilus CNRZ1066	Core299768_3	Streptococcus thermophilus CNRZ1066
Streptomyces coelicolor A3(2)	Core100226_1	Streptomyces coelicolor A3(2)
Symbiobacterium thermophilum IAM 14863	Core292459_1	Symbiobacterium thermophilum IAM 14863
Thermotoga maritima MSB8	Core243274_1	Thermotoga maritima MSB8
Thiobacillus denitrificans ATCC 25259	Core292415_3	Thiobacillus denitrificans ATCC 25259
Thiomicrospira crunogena XCL-2	Core39765_1	Thiomicrospira crunogena XCL-2
Thiomicrospira denitrificans ATCC 33889	Core326298_3	Sulfurimonas denitrificans DSM 1251
Treponema pallidum subsp. pallidum str. Nichols	Core243276_1	Treponema pallidum pallidum Nichols
Tropheryma whipplei str. Twist	Core203267_1	Tropheryma whipplei Twist
Ureaplasma parvum serovar 3 ATCC 700970	Core273119_1	Ureaplasma parvum sv 3, ATCC 700970
Vibrio cholerae O1 biovar eltor str. N16961	Core243277_1	Vibrio cholerae O1 bv El Tor, N16961
Vibrio parahaemolyticus RIMD 2210633	Core223926_1	Vibrio parahaemolyticus RIMD 2210633
Vibrio vulnificus YJ016	Seed196600_1	Vibrio vulnificus YJ016
Wolbachia sp. endosymbiont of Drosophila melanogaster	Core163164_1	Wolbachia endosymbiont of Drosophila melanogaster
Xylella fastidiosa 9a5c	Core160492_1	Xylella fastidiosa 9a5c
Yersinia pestis CO92	Core214092_1	Yersinia pestis CO-92
Zymomonas mobilis subsp. mobilis ZM4	Core264203_3	Zymomonas mobilis mobilis ZM4
Campylobacter jejuni subsp. jejuni 84-25	Core360110_3	Campylobacter jejuni jejuni 84-25
Campylobacter jejuni subsp. jejuni CF93-6	Core360111_3	Campylobacter jejuni jejuni CF93-6
Agrobacterium tumefaciens str. C58	Core176299_3	Agrobacterium tumefaciens C58-UWash
Corynebacterium glutamicum ATCC 13032	Core196627_4	Corynebacterium glutamicum Nakagawa ATCC 13032
Bartonella bacilliformis KC583	Core360095_3	Bartonella bacilliformis KC583
Francisella tularensis subsp. novicida U112	Core401614_5	Francisella tularensis novicida U112
Rhizobium leguminosarum bv. viciae 3841	Core216596_1	Rhizobium leguminosarum bv. viciae 3841
Acinetobacter baumannii ATCC 17978	Core400667_4	Acinetobacter baumannii ATCC 17978
Burkholderia cepacia R1808	Core269482_1	Burkholderia vietnamiensis G4
Shigella dysenteriae M131649	Core216598_1	Shigella dysenteriae Sd197
Yersinia pestis Pestoides F	Core386656_4	Yersinia pestis Pestoides F
Clostridium beijerincki beijerinckii NCIMB 8052	Core290402_34	Clostridium beijerinckii NCIMB 8052
Kineococcus radiotolerans SRS30216	Core266940_1	Kineococcus radiotolerans SRS30216
Klebsiella pneumoniae MGH 78578	Core272620_3	Klebsiella pneumoniae pneumoniae MGH78578
Pseudomonas putida GB-1	Core76869_3	Pseudomonas putida GB-1
Thermoanaerobacter sp. X514	Core399726_4	Thermoanaerobacter sp. X514
Elusimicrobium minutum Pei191	Core445932_3	Elusimicrobium minutum Pei191

Flavobacterium johnsonia johnsoniae UW101	Core376686_6	Flavobacterium johnsoniae UW101, ATCC 17061
Bacillus subtilis subsp. subtilis str. 168	Opt224308_1	Bacillus subtilis subtilis 168
Escherichia coli K-12, MG1655	Core83333_1	Escherichia coli K-12, MG1655
Vibrio cholerae O395	Core345073_6	Vibrio cholerae O395
Listeria monocytogenes J0161	Core393130_3	Listeria monocytogenes J0161, FSL R2-499
