# Supplementary material for: Emergent Biosynthetic Capacity in Simple Microbial Communities
Source: PLoS Comput Biol. 2014 Jul 3;10(7):e1003695. doi: 10.1371/journal.pcbi.1003695 (PMC4084645; doi:10.1371/journal.pcbi.1003695)
Supplement: Text S1 — Supporting information, including a derivation of equation (6), an analysis of the impact of emergent biosynthetic capacity on growth, an analysis of the role of the partner in emergent biosynthetic capacity, and a validation of the Goldilocks principle in universally neutral media. (PDF) [file pcbi.1003695.s012.pdf]

Supporting Text

Derivation of equation (6)

Here we explain in detail how to derive equation (6) by integrating the differential equation $dx^j/dt = \sum v_k^j * bio_k(t_1)$ over time. We start with a single species form to illustrate the basic concept. To further simplify the notation, in this derivation we drop the subscript and superscript indications (e.g., $x^j \rightarrow x$, $v_k^j \rightarrow v$, $\mu_k \rightarrow \mu$) and use B to denote the biomass (*i.e.*, $bio_k(t) \rightarrow B(t)$). With this notation, eq(6) in the single species form is as follows:

$$x(t_2) = x(t_1) + \frac{v}{\mu}\left[B(t_1)(e^{\mu\Delta t} - 1)\right]$$

This single species form of eq(6) is derived by integrating the differential equation of metabolite concentration:

$$dx/dt = v*B(t)$$

$$\rightarrow dx = v*B(t)*dt$$

The biomass B is a function of time t following exponential growth (with $B_0 \equiv B(0)$ denoting biomass at time 0), where $B(t) = B_0*e^{\mu\Delta t}$ and v is the exchange rate of metabolite x during the time interval Δt. Integrating both sides of the differential equation over time, we get:

$$\int dx = \int v*B(t)dt$$

$$= \int v*B_0*e^{\mu\Delta t}\,dt$$

$$= v*B_0\int e^{\mu\Delta t}\,dt$$

Thus:

$$x + k = v*B_0\frac{1}{\mu}e^{\mu\Delta t} \qquad (a)$$

where k is the constant of integration. Since x is a function of t, (a) becomes:

$$x(t) = \frac{v*B_0}{\mu}e^{\mu\Delta t} - k \qquad (b)$$

At $t_0 = t(0)$ we therefore get:

$$x(0) = \frac{v*B_0}{\mu}e^0 - k$$

$$\rightarrow x(0) - \frac{v*B_0}{\mu} = -k \qquad (c)$$

Taking (c) into (b) we get:

$$x(t) = \frac{v * B_0}{\mu} e^{\mu \Delta t} - k$$

$$= \frac{v * B_0}{\mu} e^{\mu \Delta t} + [x(0) - \frac{v * B_0}{\mu}]$$

$$= x(0) + \frac{v}{\mu} B_0 [e^{\mu \Delta t} - 1] \quad (d)$$

Let $t_1 = 0$, $t_2 = t$ as the beginning and end time points for the interval $\Delta t = t_2 - t_1 = t$. Substituting t with t_2, 0 with t_1, and $B_0 = B(0) = B(t_1)$ in (d), we get:

$$x(t_2) = x(t_1) + \frac{v}{\mu} [B(t_1)(e^{\mu \Delta t} - 1)] \quad (e)$$

Finally, using $bio_k(t_1)$ instead of $B(t_1)$ to generalize (e) at the community level, considering all species k, and noting the metabolite index j as superscript, we get the exact form of equation (6):

$$x^j(t_2) = x^j(t_1) + \sum_k \frac{v_k^j}{\mu_k} \left[bio_k(t_1)(e^{\mu_k \Delta t} - 1) \right] \quad \ldots (6)$$

The impact of emergent biosynthetic capacity on growth

As discussed in the main text, the main goal of our study is to characterize the prevalence of emergent biosynthetic capacity and the mechanisms that give rise to such capacity in simple communities, rather than the impact of emergent capacity on microbial growth. This is also reflected in our focus on neutral media, in which both species can successfully grow in isolation. It is interesting, however, to examine whether this metabolic capacity has a systematic impact on the overall growth of the two species in co-culture. To this end, we compared the total biomass (measured as the number of cells) of each species in co-culture at the end of each simulation to its biomass in the mono-culture, across all species pairs and media. Notably, considering our dynamics-based framework, using the total biomass as a measure of growth allowed us to estimate the overall impact of various growth conditions, whereas the maximal growth rate may only reflect a transient pattern. Interestingly, while in the large majority of cases (71.7%) growth in the co-culture clearly resulted in a growth benefit, we also found a wide range of biomass ratios, with many cases in which growth in the co-culture resulted in a marked drop in the total biomass produced (Figure S8A). This pattern is still observed when considering only

community/media combinations that exhibited emergent biosynthetic capacity and when separately examining the growth benefit of producers and partners (Figure S8B-C). Surprisingly, however, when comparing the total biomass of the community (*i.e.*, the sum of the biomass of the two species in co-culture) to the combined biomass of the two mono-cultures, a clear growth advantage is observed in the vast majority of communities (>99%; Figure S8D). In other words, while individual species may grow less successfully in co-culture (e.g., due to competition with a second, fast growing species), overall, the community benefits from the co-culture conditions. This finding is especially interesting considering our modeling framework, which in fact did not assume a community-level objective and instead allowed each species to maximize its own growth.

The role of the partner in emergent biosynthetic capacity

The role of the partner in promoting emergent secretion by the producer can involve two fundamentally different mechanisms. In one case, the partner may provide some essential cross-feeding metabolites, without which the producer is incapable of synthesizing and secreting the emergent metabolite. In such scenarios, the partner may in fact open metabolic pathways that were not accessible to the producer when grown in isolation. Conversely, by changing the composition of the medium, the partner may only modulate the cost associated with the activation of various pathways that the producer already had, allowing it to shift its metabolic activity toward a pattern that was simply suboptimal when growing in isolation.

To distinguish between these two mechanisms and to better understand the role of the partner in promoting emergent capacity, we set out to examine whether the secretion of an emergent metabolite can be achieved by the producer also in mono-culture but with some growth penalty. Specifically, for every case of emergent capacity observed in co-culture, we recorded the producer (defined as in Figure S1), the emergent metabolite, and the secretion rate of this metabolite when it was first detected. We then used standard FBA to predict the maximal growth rate of the producer in mono-culture, adding a constraint that forces the producer to secrete the emergent metabolite at the same rate as was observed in co-culture. We finally compared this obtained growth rate to the predicted growth rate of the producer in mono-culture when this constraint is not included. We found that in only 2% of the cases, the producer was not able to

grow once the constraint forcing it to secrete the emergent metabolite was added (Figure S9), indicating that a partner is essential for emergent biosynthesis and that cross-feeding is required. In all other cases, however, the producer was able to secrete the emergent metabolite in mono-culture but was consequently exhibiting suboptimal growth (compared to its growth without this constraint). This growth penalty was often relatively small, with 73% of the cases resulting in a < 5% growth decrease and 38% of cases resulting in a <1% growth decrease (Figure S9). This finding highlights the potential role of the partner not only in providing metabolic capabilities that the producer may be lacking but also in modifying the growth objective landscape and reducing the cost of previously unfavorable metabolic activity.

The Goldilocks principle in universally neutral media

The Goldilocks principle reported in the main text was demonstrated by growing each of the species pairs on 100 randomly selected, minimal and neutral media. Notably, the set of media used was different for each species pair as these media were obtained from a study that focused on pairwise species interaction and that therefore generated pairwise neutral media [1]. However, since both the community composition and the medium could impact the activity of each species in the co-culture, it may be hard to disentangle the effect of the partner on the producer's biosynthetic activity from the effect of the growth medium. We therefore wished to confirm that the Goldilocks principle holds when all species pairs are grown on the same set of media. Yet, to focus on emergence of biosynthetic capacity that is not simply a trivial byproduct of obligate symbiosis (as was our focus in the main analysis), these media should allow *each* of the six species included in our analysis to grow in mono-culture – a feature we refer to here as '*universally neutral*'. Ideally, these universally neutral media should also be minimal, promoting species interaction and increasing the likelihood of finding emergent metabolites that are not included in the initial medium. Unfortunately, however, both the method introduced in [1] for identifying minimal pairwise neutral media for the six manually curated species, and the MILP-based method we used for identifying minimal pairwise neutral media for the SEED models, could not conceptually and computationally scale up and were not suitable for identifying minimal, universally neutral media for more species.

To address this challenge, we therefore took a different approach and generated a set of 500 universally neutral media, using combinations of pairwise neutral minimal media analyzed in the main text. Specifically, to generate each such universally neutral medium, we first randomly partitioned the six species into three pairs. We then randomly selected for each pair one of the 100 pairwise neutral minimal media obtained from ref [1]. We finally combined these three media, using the union of all the nutrients comprising each medium. The resulting combined medium is universally neutral as each of the 6 species was able to grow in isolation in at least one of the three pairwise neutral media selected. This combined medium can also be conceived as 'almost' minimal as it combines three minimal media. It is however not guaranteed to be truly minimal as the combination of media may give rise to some non-trivial redundancies, and a potentially smaller universally neutral media (in terms of the number of compounds) may exist. The obtained universally neutral media contained 25-65 compounds, with a median size of ~40.

Repeating the analysis described in the main text with this set of 500 media, we again found a Goldilocks principle similar to the one shown in Figure 7A, with higher levels of emergent capacity observed when the species comprising the community are functionally neither too close, nor too distant (Figure S10). Overall, the number of emergent metabolites observed with these media was somewhat lower (compare, for example, Figure 7A and Figure S10), potentially due to the fact that these media are not in fact minimal and may therefore exert less pressure for species interaction and for emergent metabolism. Yet, the numbers of emergent metabolites in the functionally intermediate species pairs were still significantly higher than those observed in both the functionally close species group and the functionally distant species group ($p<10^{-57}$; two sample t-test). A similar significant pattern was obtained when using phylogenetic rather than functional distance to measure the similarity between species.

References

1. Klitgord N, Segrè D (2010) Environments that induce synthetic microbial ecosystems. PLoS computational biology 6: e1001002. doi:10.1371/journal.pcbi.1001002.
